# Genetic diversity and evolution of porcine hemagglutinating encephalomyelitis virus in Guangxi province of China during 2021–2024

**DOI:** 10.3389/fmicb.2024.1474552

**Published:** 2024-10-09

**Authors:** Kaichuang Shi, Xin Hu, Feng Long, Yuwen Shi, Yi Pan, Shuping Feng, Zongqiang Li, Yanwen Yin

**Affiliations:** ^1^School of Basic Medical Sciences, Youjiang Medical University for Nationalities, Baise, China; ^2^College of Animal Science and Technology, Guangxi University, Nanning, China; ^3^Guangxi Center for Animal Disease Control and Prevention, Nanning, China

**Keywords:** coronavirus, porcine hemagglutinating encephalomyelitis virus, phylogenetic analysis, genetic evolution, recombination

## Abstract

Porcine hemagglutinating encephalomyelitis virus (PHEV) is the only known porcine neurotropic coronavirus, which is prevalent worldwide at present. It is of great significance to understand the genetic and evolutionary characteristics of PHEV in order to perform effective measures for prevention and control of this disease. In this study, a total of 6,986 tissue samples and nasopharyngeal swabs were collected from different regions of Guangxi province in southern China during 2021-2024, and were tested for PHEV using a quadruplex RT-qPCR. The positivity rate of PHEV was 2.81% (196/6,986), of which tissue samples and nasopharyngeal swabs had 2.05% (87/4,246) and 3.98% (109/2,740) positivity rates, respectively. Fifty PHEV positive samples were selected for PCR amplification and gene sequencing. Sequence analysis revealed that the nucleotide homology and amino acid similarities of S, M, and N genes were 94.3%-99.3% and 92.3%-99.2%, 95.0%-99.7% and 94.7%-100.0%, 94.0%-99.5% and 93.5%-99.3%, respectively, indicating M and N genes were more conservative than S gene. Phylogenetic trees based on these three genes revealed that PHEV strains from different countries could be divided into two groups G1 and G2, and the PHEV strains from Guangxi province obtained in this study distributed in subgroups G1c and G2b. Bayesian analysis revealed that the population size of PHEV has been in a relatively stable state since its discovery until it expanded sharply around 2015, and still on the slow rise thereafter. S gene sequences analysis indicated that PHEV strains existed variation of mutation, and recombination. The results indicated that the prevalent PHEV strains in Guangxi province had complex evolutionary trajectories and high genetic diversity. To the best of our knowledge, this is the first report on the genetic and evolutionary characteristics of PHEV in southern China.

## 1 Introduction

Porcine hemagglutinating encephalomyelitis (PHE) is a viral disease of pigs caused by PHE virus (PHEV). PHEV, which belongs to *coronaviridae* family, *Betacoronavirus* genus, *Embecovirus* subgenus, is the only known porcine neurotropic coronavirus (Turlewicz-Podbielska and Pomorska-Mól, 2021; Llanes et al., 2020). PHEV is a large enveloped, single-stranded positive-sense RNA virus, with approximately 30 kb genome containing at least 11 open reading frames (ORFs) (Shi et al., 2018). The first two ORFs encode 16 non-structural proteins (NSP1-NSP16), and the rest of ORFs encode structural proteins including surface spike glycoprotein (S), transmembrane glycoprotein (M), nucleocapsid protein (N), and membrane protein (E), and auxiliary proteins including NS2, NS4.9, NS12.7, and N2 proteins (Shi et al., 2018). In addition, PHEV also has a glycoprotein encoded by ORF3 related to envelope, which is called hemagglutinin-esterase (HE). Due to this protein, PHEV has hemagglutination properties that other porcine coronaviruses do not have (Sasseville et al., 2002). S, M, and N proteins play important roles in coronaviruses. S protein belongs to class I fusion protein (Bosch et al., 2003), a trimer structure, that can be fixed on the viral envelope and decorates the surface of the virus particles with its outer domain, which is the reason why coronavirus has a unique coronal spinous process structure (Beniac et al., 2006). In the key process of coronavirus infecting host, S protein, which contains main antigens and antiviral neutralization determinants (Mora-Díaz et al., 2019), can modify the surface of virions, induce neutralizing antibody response, and promote virus invasion into host cells and transmission in the host through interaction with HE protein (Cornelissen et al., 1997). These functions make S protein play an important role in the development, diagnosis, and treatment of coronavirus vaccine (Zhang et al., 2021). In addition, some regions of S protein can interact with nerve cell adhesion molecule (NCAM) expressed on the surface of neurons, which makes S protein play an important role in the process of PHEV infecting neurons (Dong et al., 2015; Gao et al., 2010). M protein is the most abundant protein in coronavirus particles (Armstrong et al., 1984). The various functions of M protein in the viral infection cycle and interferon antagonism make it the most conservative and constrained structural protein of the virus (Cagliani et al., 2020). M protein can adopt two different conformations, an elongated one and a compact one (Neuman et al., 2011), which are related to different properties. The appropriate proportion of these two conformations also determines the final viral particle structure. In addition to its affinity to itself, M protein can bind to S protein, N protein, E protein, and genomic RNA. This is because it has both homotypic and heterotypic associative properties (Wong and Saier, 2021), so M protein also plays an important role as an adhesive in virus assembly (Wong and Saier, 2021; Arndt et al., 2010). N protein is the most abundant coronavirus antigen produced during infection (Li et al., 2003), and it is also the only nucleocapsid protein that interacts with viral RNA to form a spiral ribonucleoprotein complex (Mora-Díaz et al., 2019). It plays a role in the synthesis and transcription of viral RNA and the replication and regulation of metabolism in infected cells (Cong et al., 2020; Huang et al., 2004). The most important role of N protein is that, as a capsid protein, it protects genomic RNA through packaging and maintains the stability of RNA in the virus (Gorkhali et al., 2021). The binding of N protein to leader RNA is essential for maintaining the organized RNA conformation of viral genome replication and transcription (Tang et al., 2005; Stohlman et al., 1988). Some studies have also shown that the comparative study of N proteins of different coronaviruses can provide valuable information for host specificity and the interaction between N proteins and host cell proteins (McBride et al., 2014; Emmott et al., 2013). These provide new insights into the development of new antiviral therapies for the interaction between host cell proteins and N proteins (Meyniel-Schicklin et al., 2012). Therefore, S, M, and N genes are usually taken as the target genes for epidemiological study due to their important roles in coronaviruses.

PHE was first discovered in Ontario, Canada in 1957 (Roe and Alexander, 1958). Piglets infected with PHEV showed vomiting, anorexia, constipation, and severe progressive weight loss. Then, it was systematically reported that infected newborn piglets developed anorexia, trembling, curling, and vomiting, followed by ataxia, hyperactivity, slapping, and other neurological symptoms, and died 2–3 days after the appearance of clinical symptoms (Alexander et al., 1959; Mitchell et al., 1961). Pigs are the only naturally infected host of PHEV, and PHEV is the only known neurotropic coronavirus in pigs. Besides, mice and Wistar rats artificially infected with PHEV also show neurological symptoms (Hirano et al., 2004; Yagami et al., 1993). PHEV can infect pigs of all ages, but the clinical symptoms after infection are not only related to the virulence of the virus, but also related to the age of infected pigs. The morbidity and mortality of infected pigs are closely related to their ages (Mora-Díaz et al., 2021). For growing and adult pigs, PHEV infection is subclinical because it induces strong humoral immune response (Lorbach et al., 2017; Quiroga et al., 2008), while for newborn piglets, PHEV infection is fatal. According to clinical symptoms, PHE can be divided into two types: encephalomyelitis type and vomiting exhaustion type. When newborn piglets are infected with PHEV, it invades the nervous system, resulting in sneezing, coughing, vomiting, and other nervous system symptoms, including ataxia, muscle tremor, hyperesthesia, and finally dyspnea, side lying, coma, and then death. The mortality rate of newborn piglets infected with PHEV is almost up to 100% (Mora-Díaz et al., 2019). The main symptoms of vomiting exhaustion are digestive tract symptoms, including vomiting, diarrhea, weight loss, and so on (Turlewicz-Podbielska and Pomorska-Mól, 2021; Mengeling and Cutlip, 1972; Cutlip and Mengeling, 1972). It is also worth noting that the elderly pigs infected with PHEV developed respiratory symptoms reported at an exhibition in 2015 (Lorbach et al., 2017). There were reports that the main route of PHEV infection was through the respiratory epithelium, and sneezing and coughing were the first symptoms that might be observed in the infected pigs, indicating the effects of PHEV on the upper respiratory tract and lungs of pigs (Lorbach et al., 2017; Cutlip and Mengeling, 1972; Alsop, 2006). However, the specific role of PHEV as a respiratory pathogen needs further study.

Even though PHEV is one of the first porcine coronaviruses to be discovered and isolated (Turlewicz-Podbielska and Pomorska-Mól, 2021), its harm to the pig industry has usually been ignored. Because there are no specific therapeutic drugs and commercial vaccines, PHEV has been prevalent around the world and under constant mutation. In recent years, new variants of PHEV have also been found in China, which can cause respiratory diseases in growing pigs and adult pigs (He et al., 2023). In 2015, there were cases of respiratory diseases caused by PHEV infection in adult pigs at an exhibition in Michigan, USA (Lorbach et al., 2017). Recently, a strain of PHEV-causing diarrhea in pigs was also isolated in South Korea (Kim et al., 2022). All these events indicated that PHEV has been constantly mutating in the process of evolution. PHEV has been found in Guangxi province, southern China (Hu et al., 2023). In this study, the clinical tissue samples (including brain, lung, liver, and spleen of each pig) and nasopharyngeal swabs were collected from pig farms, harmless treatment plants, and slaughterhouses in Guangxi province during 2021–2024, and tested for PHEV using a quadruplex real-time quantitative RT-PCR (RT-qPCR) (Hu et al., 2023). The PHEV-positive samples were selected to amplify S, M, and N genes, and then sequence and analyze in order to understand the genetic characteristics and evolution of PHEV in Guangxi province. To our best knowledges, this is the first report on the genetic and evolutionary analysis of PHEV in southern China.

## 2 Materials and methods

### 2.1 Detection of clinical samples

From January 2021 to January 2024, 4,246 tissue samples (including brain, lymph nodes, lung, and spleen of each pig, and the tissue homogenate from each pig was considered as one sample when tested), and 2,740 nasopharyngeal swabs were collected from pig farms, slaughterhouses, and harmless treatment plants in 14 regions in Guangxi province, southern China. After collection, the samples were transported to our laboratory under ≤4°C within 8 h, and then stored at −80°C until use.

The clinical tissue samples (0.05 g of brain, lymph node, lung, and spleen each) were put into 2.0 mL EP tube, added 1.0 mL phosphate buffer solution (PBS, pH7.2, W/V = 1:5) and a sterilized steel ball, ground 5 min in an oscillatory grinder, frozen and thawed 3 times, and then centrifuged at 4°C (12 000 rpm for 5 min) to obtain the supernatant. The nasopharyngeal swabs contained in 2.0 mL EP tube were put into 1.0 mL PBS (pH7.2), vibrated 30 s, frozen and thawed 3 times, and then centrifuged at 4°C (12 000 rpm for 5 min) to obtain the supernatant. Two hundred microlitter supernatants of tissue samples and nasopharyngeal swabs were used to extract total nucleic acids using MiniBEST Viral DNA/RNA Nucleic Acid Extraction Kit Ver.5.0 (TaKaRa, Dalian, China). The extracted nucleic acids were stored at −80°C until use.

The total DNA/RNA of clinical samples were tested for PHEV using a quadruplex RT-qPCR developed in our laboratory (Hu et al., 2023). Then, 50 PHEV-positive samples were selected for sequence analysis. The total DNA/RNAs were extracted from the PHEV-positive samples, reverse transcribed into cDNA using PrimeScript™ II 1st Strand cDNA Synthesis Kit (TaKaRa, Dalian, China), and used to amplify and sequence S, M, and N genes of PHEV.

### 2.2 Amplification of S, M, and N genes

Based on PHEV genome sequence downloaded from the National Center for Biotechnology Information (NCBI, https://www.ncbi.nlm.nih.gov/) (GenBank accession number: KY419112.1), specific primers were designed to amplify S, M, and N genes of PHEV (Table 1). The cDNAs of PHEV-positive samples were used as templates to amplify S, M, and N gene fragments using the designed primers (Table 1). The PCR amplification system was as follows: Premix Taq (Ex Taq Version 2.0 plus dye) (TaKaRa, Dalian, China) 25 μL, forward/reverse primer (20 μM) 1 μL each, cDNA 5 μL, nuclease-free distilled water up to total volume of 50 μL. The amplification procedures were as follows: S1 gene fragment: 35 cycles of 95°C 15 s, 61°C 30 s, and 72°C 30 s, then 72°C 10 min; S2, S3, S4, S5, N1, and N2 gene fragments: 35 cycles of 95°C 15 s, 57°C 30 s, and 72°C 30 s, then 72°C 10 min; M gene fragment: 35 cycles of 95°C 15 s, 54°C 30 s, and 72°C 30 s, then 72°C 10 min.

**Table 1:** Primers for amplification of PHEV S, M, and N genes.

Gene	Primer	Sequence (5′ → 3′)	Product size/bp
S	PHEV-S1-F	GCCCTACTGCTGCTAGTATTATT	868
	PHEV-S1-R	GGTCACAAAACCAGTATCTGT	
	PHEV-S2-F	CAGATACTGGTTTTGTGACCAAG	948
	PHEV-S2-R	CTTTRGATTGCGGACAAGTCC	
	PHEV-S3-F	AGAGGCCTTCATGATGCTGT	905
	PHEV-S3-R	GTAAAGCGATAACCTGTAGT	
	PHEV-S4-F	CAGCTAGTGCTGTAAGTACT	1,099
	PHEV-S4-R	TCACTAAGCTGCTGAGAAAC	
	PHEV-S5-F	GGCTGTTGTTAATGCAAATGC	971
	PHEV-S5-R	GACGAAATTAATCGTCATGTG	
M	PHEV-M-F	TGTGTATTCAACTTTGCGGTATG	902
	PHEV-M-R	GATTTCCAGAGGACGCTCTAC	
N	PHEV-N1-F	GGACACCGCATTGTTGAGAAATA	767
	PHEV-N1-R	TTGCCAGAACGAGACTAGCAA	
	PHEV-N2-F	CGGTACTCCCTCAAGGTTACTA	876
	PHEV-N2-R	GAGTGCCTTATCCCGACTTTC	

R = A/G.

### 2.3 Sequencing of S, M, and N genes

The PCR products were purified using MiniBEST DNA Fragment Purification Kit Ver.4.0 (TaKaRa, Dalian, China), ligated into pMD18-T vector (TaKaRa, Dalian, China), and then transformed into *E. coli.* DH5α competent cells (TaKaRa, Dalian, China). The positive clones were inoculated in LB medium containing ampicillin, cultured at 37°C for 20–24 h, and sequenced by IGE Biotechnology LTD (Guangzhou, China). The sequences obtained by sequencing was spliced using the EditSeq tool in Lasergene DNAstar 7.0 software^1^ to obtain the complete gene sequences of S, M, and N genes. The obtained complete gene sequences were aligned using the BLAST tool at NCBI.^2^

### 2.4 Sequence analysis of S, M, and N genes

The obtained S, M, and N gene sequences in this study were compared with 51 S gene sequences, 48 M gene sequences, and 54 N gene sequences of PHEV downloaded from NCBI (Supplementary Tables S1–S3), which were collected from China, America, Canada, Korea, Belgium, the Netherlands, and the Czech Republic. The similarities of nucleotides and amino acids among the obtained sequences and reference sequences were analyzed using Clustal W algorithm of Bioedit software.^3^ The best DNA/protein models of S, M, and N genes were found using MEGA.X software.^4^ According to the optimal nucleotide models, the phylogenetic trees of S, M, and N genes were constructed using MEGA.X based on the maximum likelihood (ML) method, and then optimized through the online website Interactive Tree of Life (iTOL)^5^ (Letunic and Bork, 2021).

### 2.5 Bayesian temporal dynamics analysis of S gene

A total of 101 S gene sequences, including 50 sequences obtained in this study and 51 sequences downloaded from NCBI (Supplementary Table S1), were aligned using the MEGA.X software (see text footnote 4) The root-to-peak genetic distances of these sequences were determined using the TempEst v1.6 in BEAST v1.10.4 software^6^ (Rambaut et al., 2016) to determine the temporal structure (Tsai et al., 2015). The divergence times of PHEV strains in BEAST v1.10.4 software (see text footnote 6) were derived using Bayesian Markov chain Monte Carlo (MCMC) approach (with the strict clock), Bayesian skyline coalescent, and the optimal nucleotide substitution model (Fan et al., 2022; Siah et al., 2020). Then, the MCMC was run in parallel on 3 chains with 200 million steps per chain and a burn-in of 10%. Convergence was visually confirmed for all parameters (ESS > 200) by Tracer v1.6 in BEAST v1.10.4 software (see text footnote 6) (Zhao et al., 2016). The maximum clade credibility (MCC) tree was obtained using Tree Annotator in BEAST v1.10.4 software (see text footnote 6), and visualized through Figtree v1.4.4 (see text footnote 6) (Woo et al., 2016).

### 2.6 Genome recombination events analysis

The S amino acid sequences of 50 PHEV strains obtained in this study were compared with those of the reference strains using BioEdit software (see text footnote 3). Then the recombination events analysis of S gene was performed on Recombination Detection Program (RDP4) software,^7^ with a window size of 500 bp and a *p*-value of 0.05. According to the manual of RDP4, seven algorithms in the RDP4 software, including RDP, GENECONV, BootScan, MaxChi, Chimaera, SiScan, and 3Seq, were selected and analyzed for the recombination event of all S gene sequences. Sequences were considered to be potentially recombinant only when at least 6 algorithms supported them, and the putative recombination events were also verified using the SimPlot software.^8^

## 3 Results

### 3.1 Detection results of clinical samples

**Figure 1:**
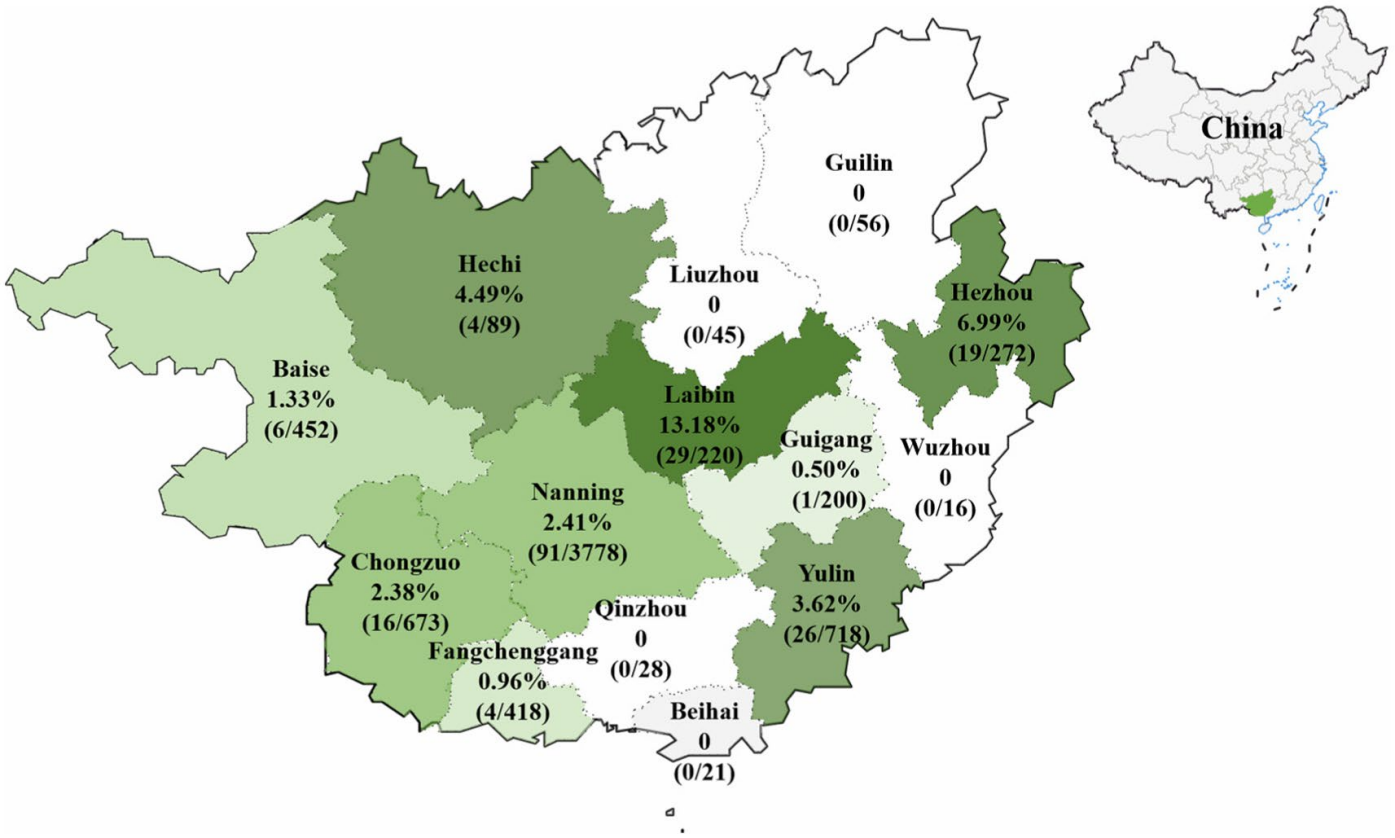
The distribution of PHEV-positive samples in Guangxi province, southern China.

The 6,986 clinical samples (4,246 tissue samples and 2,740 nasopharyngeal swabs) collected from 14 regions in Guangxi province during 2021–2024 were tested for PHEV using a quadruplex RT-qPCR (Hu et al., 2023). The PHEV positivity rate in clinical samples was 2.81% (196/6,986), of which tissue samples and nasopharyngeal swabs had 2.05% (87/4,246) and 3.98% (109/2,740) positivity rates, respectively (Supplementary Table S4). Of 14 regions in Guangxi province, Laibin had the highest positivity rate of 13.18%, while no positive sample was found in samples from 5 regions, i.e., Guilin, Liuzhou, Wuzhou, Qinzhou, and Beihai. The distribution of the positive samples in Guangxi province is shown in Figure 1.

### 3.2 Amplification and sequencing of S, M, and N genes

Of the 196 PHEV positive samples, 50 samples were selected basing on sampling site, sampling date, and Ct values, and used for gene amplification, and sequence analysis. The target fragments of S, M, and N genes were amplified using the specific primers (Table 1). After purification, ligation, transformation, sequencing, and splicing, 50 S, 50 M, and 50 N gene sequences of PHEV strains were obtained. The gene sequences were uploaded to NCBI GenBank under the accession numbers: PP646298-PP646347 for S gene, PP646348-PP646397 for M gene, and PP646398-PP646447 for N gene. The information on these gene sequences is shown in Supplementary Tables S1–S3.

### 3.3 Similarity analysis of S, M, and N genes

The S gene nucleotide and amino acid sequences obtained in this study and reference strains were analyzed using Clustal W algorithm of BioEdit software. The results revealed that the nucleotide and amino acid similarities of S, M, and N genes among 50 PHEV strains obtained in the study were 94.5–99.8% and 92.5–99.6%, 94.6–100.0% and 93.4–100.0%, and 96.8–100% and 95.9–100.0%, respectively. The nucleotide and amino acid similarities among 50 strains and reference strains of 51 S, 48 M, and 54 N genes were 94.3–99.3% and 92.3–99.2%, 95.0–99.7% and 94.7–100.0%, and 94.0–99.5% and 93.5–99.3%, respectively (Table 2).

**Table 2:** The similarity analysis of S, M, and N gene sequences.

Gene	Similarity among the strains obtained in this study		Similarity among the strains obtained in this study and the reference strains	
	Nucleotide (%)	Amino acid (%)	Nucleotide (%)	Amino acid (%)
S	94.5–99.8	92.5–99.6	94.3–99.3	92.3–99.2
M	94.6–100.0	93.4–100.0	95.0–99.7	94.7–100.0
N	96.8–100.0	95.9–100.0	94.0–99.5	93.5–99.3

### 3.4 Phylogenetic analysis of S, M, and N genes

#### 3.4.1 Phylogenetic analysis based on S gene sequences

**Figure 2:**
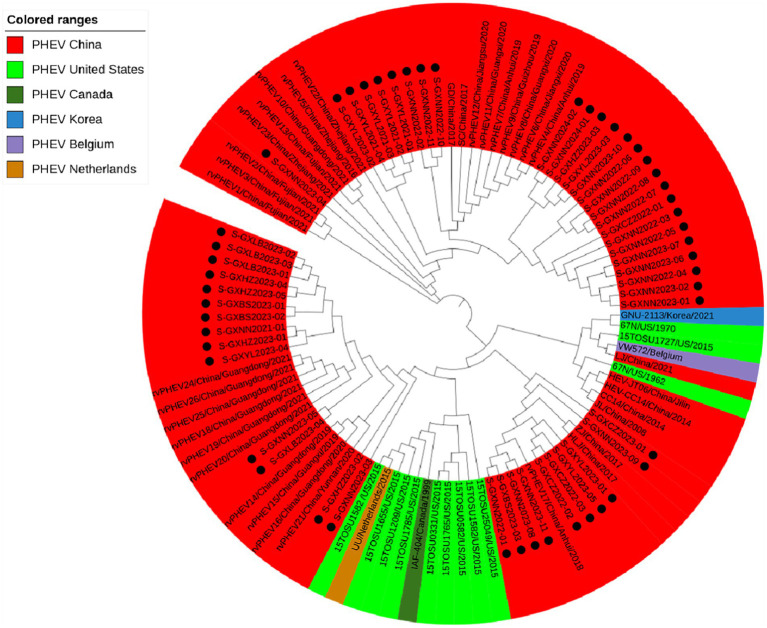
Phylogenetic tree based on PHEV S gene nucleotide sequences. The black spots indicate the PHEV S sequences obtained in this study.

Basing on the best nucleotide substitution model: TN93 + G + I, a phylogenetic tree was generated based on S gene sequences of 50 PHEV strains obtained in this study and 51 reference PHEV strains downloaded from NCBI, which was constructed using maximum likelihood (ML) after 1,000 bootstrap tests (Figure 2). The phylogenetic tree indicated that PHEV strains were classified into two groups. The first group included the strains obtained in this study (including 18 strains from Nanning, 1 strain from Chongzuo, 1 strain from Hezhou, 6 strains from Yulin), and other 17 strains collected from other several provinces in China. The second group included the strains from the United States (12 strains), China (18 strains), Canada (1 strain), Belgium (1 strain), Korea (1 strain), the Netherlands (1 strain), and 24 strains obtained in this study.

#### 3.4.2 Phylogenetic analysis based on M gene sequences

**Figure 3:**
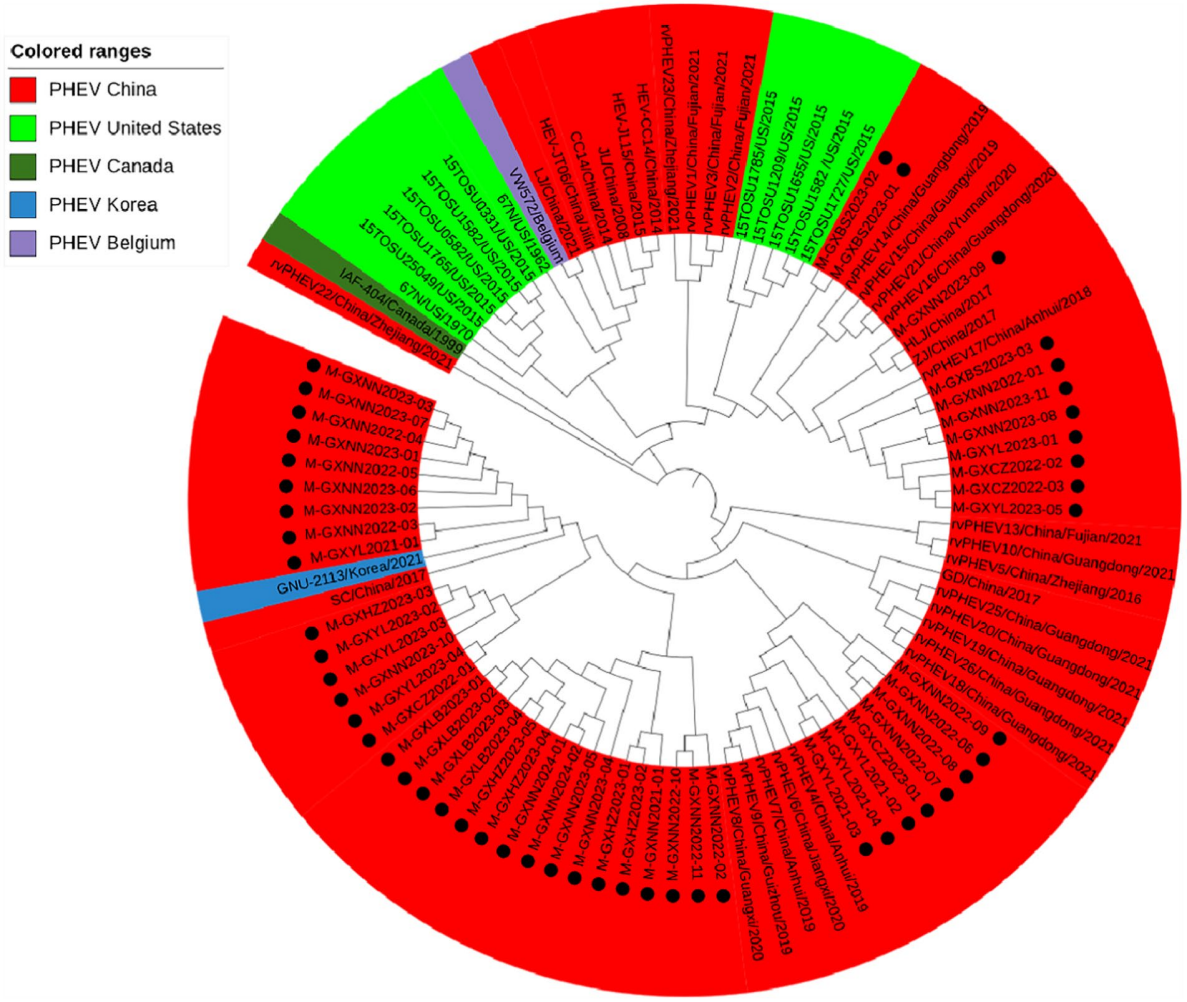
Phylogenetic tree based on PHEV M gene nucleotide sequences. The black spots indicate the PHEV M sequences obtained in this study.

Basing on the best nucleotide substitution model: TN93 + G, a phylogenetic tree was generated based on M gene sequences of 50 PHEV strains obtained in this study and 48 reference PHEV strains downloaded from NCBI, which was constructed by ML after 1,000 bootstrap tests (Figure 3). The phylogenetic tree revealed that all PHEV strains were divided into two groups. The first group included the strains from China (15 strains), Korea (1 strain), and 39 strains obtained in this study, including those from Nanning (21 strains), Chongzuo (2 strains), Hezhou (5 strains), Yulin (7 strains), and Laibin (4 strains). The second group contained the other 11 strains obtained in this study, 12 strains from the United States, 18 strains from other provinces in China, 1 strain from Canada, and 1 strain from Belgium.

#### 3.4.3 Phylogenetic analysis based on N gene sequences

**Figure 4:**
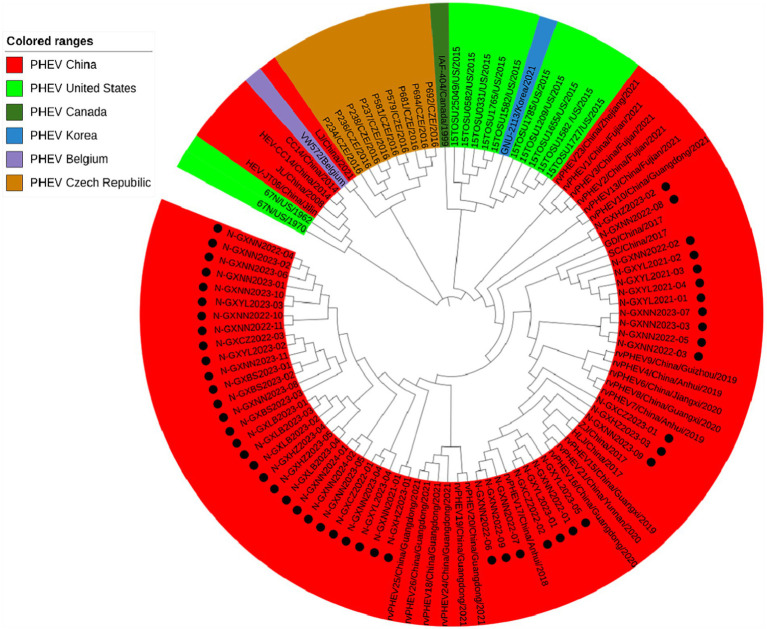
Phylogenetic tree based on PHEV N gene nucleotide sequences. The black spots indicate the PHEV N sequences obtained in this study.

Basing on the best nucleotide substitution model: GTR + G + I, a phylogenetic tree was generated based on N gene of 50 PHEV strains obtained in this study and 54 reference PHEV strains downloaded from NCBI, which was constructed by ML after 1,000 bootstrap tests (Figure 4). The phylogenetic tree indicated that all PHEV strains were divided into two groups. The first group included all 50 strains obtained in this study, and 25 strains from other provinces of China, 10 strains from the United States, 1 strain from Canada, 1 strain from Korea. The second group included strains obtained from China (5 strains), the United States (2 strains), the Czech Republic (9 strains), and Belgium (1 strain).

### 3.5 Analysis of S gene amino acid sequences

To further analyze genetic characteristics of PHEV, the S gene amino acid sequences of 50 PHEV strains obtained in this study were compared with those of the reference strains downloaded from NCBI using BioEdit software. The earliest PHEV strain 67 N (GenBank accession no. AY078417) was used as the prototype reference strain. The results showed that the 50 PHEV strains obtained in this study had multiple mutations compared with the reference strain (Table 3). Almost all of the 50 PHEV strains obtained in this study had amino acid mutations at positions 22, 169, 253, 512, 611, 804, 1,013, 1,189, and 1,332 (Supplementary Figure S1). Some of the 50 PHEV strains obtained in this study had amino acid mutations at sites 8, 25, 73, 78, 98, 101, 111, 141, 154, 352, 407, 410, 416, 552, 557, 608, 703, 705, 756, 770, 884, 959, 1,034, 1,066, 1,167, 1,198, and 1,252.

**Table 3:** Mutations of S gene amino acid sequences of PHEV strains obtained in this study.

Position	Mutation	Position	Mutation	Position	Mutation
8	S → T/F	352	I → T	770	A → S/V
22	T → N	407	V → L	804	R → S
25	L → S	410	S → F	884	P → S
73	A → S	416	F → S	959	L → S
78	M → V	512	K → N	1,013	A → S
98	P → S	552	G → A	1,034	A → S/L
101	D → H	557	D → E	1,066	A → V
111	R → K	608	G → S	1,167	I → M/T/L
141	E → K/D	611	I → N	1,189	S → G
154	L → F	703	I → V/A	1,198	Q → R
169	H → N/Q	705	R → G	1,252	I → T/N
253	N → D	756	F → V	1,332	C → F

### 3.6 Bayesian time dynamic analysis of S gene

**Figure 5:**
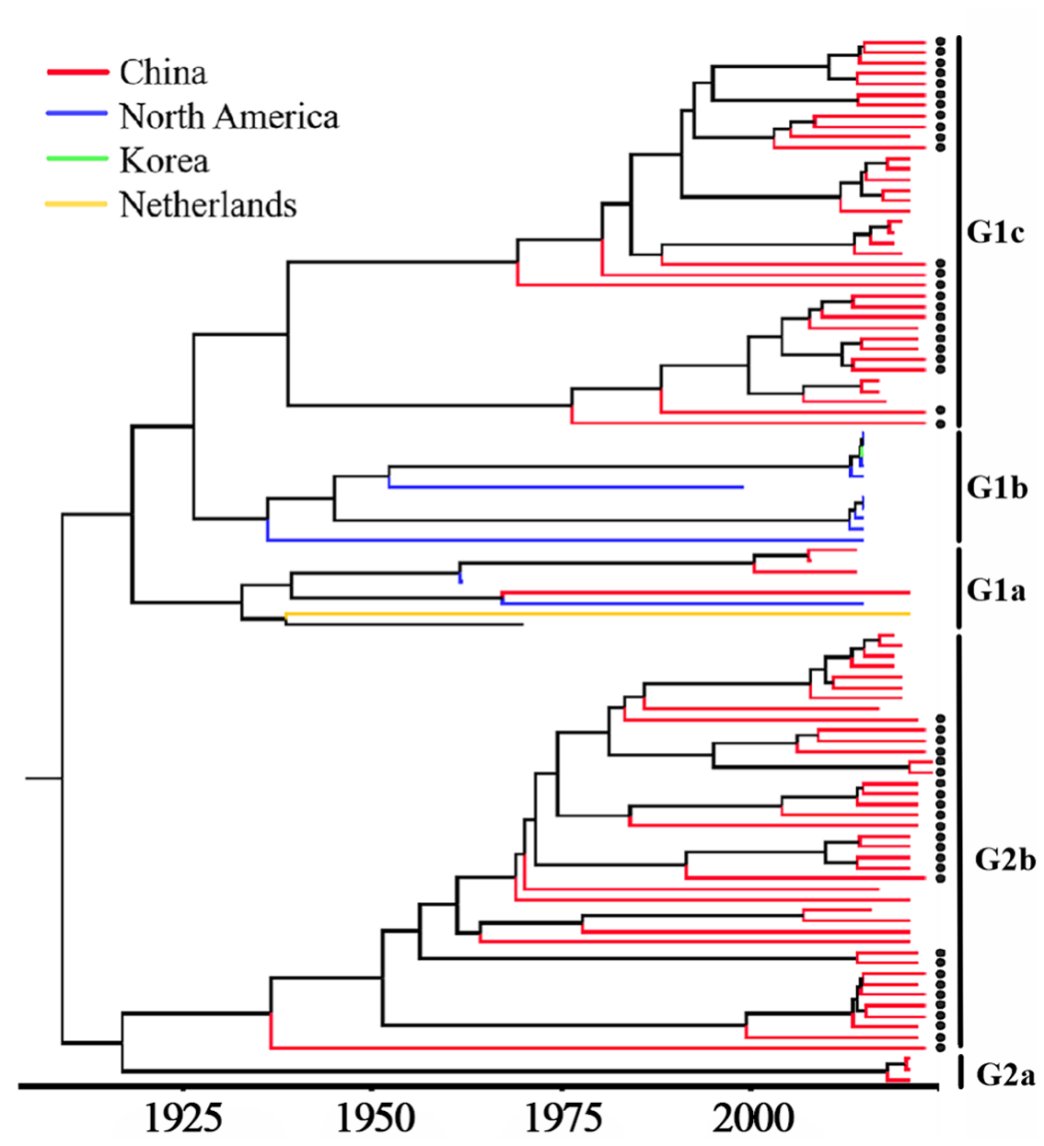
MCC tree based on PHEV S gene nucleotide sequences. The sequences obtained in this study are marked with black spots.

**Figure 6:**
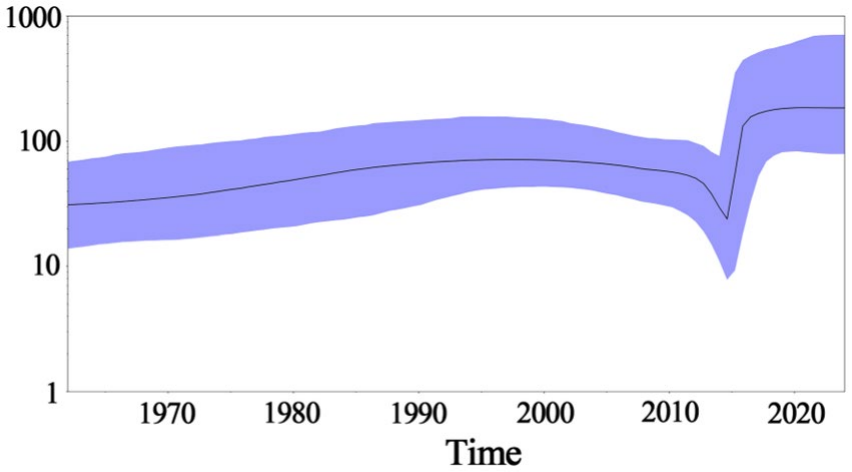
Bayesian skyline of PHEV S gene. The dark purple line indicates the average of genetic diversity, and the light purple shading indicates a 95% confidence interval.

Based on PHEV S gene sequences, the temporal scale MCC tree was constructed using BEAST v1.10.4 software and Figtree v1.4.4 (Figure 5). The results showed that PHEV strains were divided into two groups (Group 1 and Group 2), while Group 1 could be further divided into three subgroups, i.e., Group 1a, Group 1b, and Group 1c, and Group 2 could be further divided into two subgroups, i.e., Group 2a, and Group 2b. The PHEV strains obtained in this study were located in two subgroups, i.e., Group 1c and Group 2b, and they distributed in different clades, which was consistent with the phylogenetic tree constructed using MEGA.X software (Figure 2). The Bayesian skyline (Figure 6) showed the adequate population size of PHEV transmission, which has been in a relatively stable state since its discovery until it expanded sharply around 2015. On the whole, the effective population size of PHEV has been on the rise.

### 3.7 Genetic evolution rates of S, M, and N genes

The genetic evolution rates of PHEV S, M, and N genes were analyzed using BEAST v1.10.4 software. The results showed that the genetic evolution rates of S, M, and N genes were 2.655 × 10^−4^, 5.436 × 10^−4^, and 3.106 × 10^−4^ (substitution/site/year), respectively (Table 4). The results indicated that M, and N genes were more conservative than S gene, but there was no significant difference among the genetic evolution rates of S, M, and N genes.

**Table 4:** Estimation of evolution rates of S, M, and N genes.

Gene	Mean evolutionary rate (substitution/site/year)	95% HPD (substitution/site/year)
S	2.655 × 10^−4^	2.187 × 10^−4^- 3.141 × 10^−4^
M	5.436 × 10^−4^	3.322 × 10^−4^- 7.556 × 10^−4^
N	3.106 × 10^−4^	2.273 × 10^−4^- 4.006 × 10^−4^

### 3.8 Recombination analysis of S gene sequences

**Figure 7:**
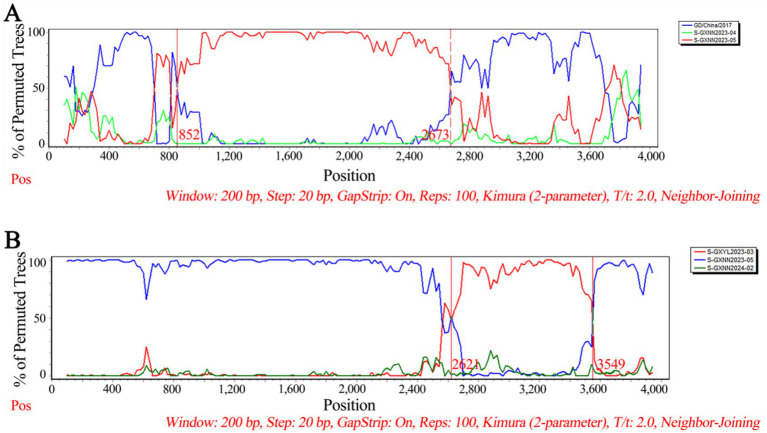
The possibility of recombination event analysis of the PHEV GXNN2023-04 strain **(A)**, and GXNN2024-02 strain **(B)**.

The recombination analysis of PHEV S gene was analyzed using RDP4.0 software, and the results were further confirmed using SimPlot analysis. The results indicated that two PHEV strains showed potential recombination events. The GXNN2023-04 strain derived from recombination between GXNN2023-05 and OQ305205.1: PHEV/GD/2017 strains, and the breakpoints for the recombination were found in 852 nt-2673 nt. The GXNN2024-02 strain derived from recombination between GXYL2023-03 and GXNN2023-05 strains, and the breakpoints for the recombination were found in 2621 nt-3549 nt (Figure 7).

## 4 Discussion

Six coronaviruses are known to infect pigs, including PHEV, transmissible gastroenteritis virus (TGEV), porcine respiratory coronavirus (PRCoV), porcine epidemic diarrhea virus (PEDV), swine acute diarrhea syndrome coronavirus (SADS-CoV), and porcine deltacoronavirus (PDCoV) (Turlewicz-Podbielska and Pomorska-Mól, 2021). In addition, the recombinant coronavirus derived from TGEV and PEDV recombinant was reported, which need to pay more attention and do further study (Boniotti et al., 2016). Since the PHEV infected adult pigs are usually subclinical, the economic loss of this disease has usually been ignored. PHEV was first discovered in Canada in 1957, and it has been reported in Europe, America, and Asia (Mora-Díaz et al., 2019; Roe and Alexander, 1958; Quiroga et al., 2008; Rho et al., 2011; Cartwright et al., 1969; Pensaert and Callebaut, 1974; Gao et al., 2011; Dong et al., 2014). In China, Gao et al. (2011) and Dong et al. (2014) reported the epidemic of encephalitis in PHEV-infected piglets. The pigs at an exhibition in Michigan in 2015 showed influenza-like diseases, and were confirmed to be infected with PHEV. The screening of all samples showed that the positivity rate of PHEV was as high as 38.7%. In addition, variants of PHEV were also found, and 10 full sequences and one partial sequence were obtained (Lorbach et al., 2017). After that, there was an acute outbreak of diarrheal disease in newborn piglets in a farm in Seoul, Korea in 2021, and the mortality rate of piglets reached 40%. It was confirmed that these diarrhea piglets were infected with PHEV (Kim et al., 2022; Li et al., 2016). In recent years, respiratory phenotypic variants of PHEV have also been found in China. He et al. (2023) confirmed the prevalence of PHEV in at least eight provinces in southeastern China through large-scale epidemiological surveillance. Twenty-four PHEV strains were analyzed, and there was extensive recombination between these respiratory phenotypic PHEV and classical PHEV and previous respiratory variants. These results indicated that PHEV has been prevalent in many countries around the world, and showed variant in pig herds.

The 6,896 clinical tissue samples and nasopharyngeal swabs from Guangxi province during 2021–2024 were detected for PHEV using a multiplex RT-qPCR (Hu et al., 2023). Fifty PHEV-positive samples were selected for amplification and sequencing S, M, and N genes, and these gene sequences were compared with the reference strains downloaded from NCBI. The nucleotide and amino acid similarities between S, M, and N genes of 50 PHEV strains obtained in this study were 94.5–99.8% and 92.5–99.6%, 94.6–100.0% and 93.4–100.0%, 96.8–100.0% and 95.9–100.0%, respectively, indicating there were genetic differences among prevalent strains. The nucleotide and amino acid similarities of S, M, and N genes between the strains obtained in this study and the reference strains were 94.3–99.3% and 92.3–99.2%, 95.0–99.7% and 94.7–100.0%, 94.0–99.5% and 93.5–99.3%, respectively, indicating significant differences between Guangxi’s strains and other domestic and abroad strains. Of the phylogenetic tree based on S gene sequences, PHEV strains were divided into two groups. Twenty-six strains of 50 PHEV strains obtained in this study had high homology with SC and GD strains isolated by Sun et al. (2023) and some Chinese respiratory variant PHEV (rvPHEV) strains newly found by He et al. (2023). The other 24 PHEV strains had high homology with 67 N strain which was first found and isolated in the United States, 10 respiratory phenotypic PHEV strains isolated in North America in 2015, Korean, Belgian and Dutch strains, and some Chinese rvPHEV strains found by He et al. (2023). The PHEV strains in the phylogenetic trees based on M and N genes were also divided into two groups, and 50 PHEV strains obtained in this study were located in two groups. Several other reports also showed that the prevalent PHEV strains could be divided in two groups based on complete genome sequence, and the phylogenetic tree based on S gene sequences could also divided into two groups and showed similar topology with those based on complete genome sequence (Shi et al., 2018; He et al., 2023; Sun et al., 2023; Bahoussi et al., 2022). It is noteworthy that the newly identified respiratory variant PHEV (rvPHEV) strains were discovered to cause exclusively respiratory symptoms (He et al., 2023), and the PHEV strains obtained in this study distributed in G1c and G2b, which were corresponded to rvPHEV-L-2 and rvPHEV-L-1 reported by He et al. (2023), indicating that all the strains obtained in Guangxi province belonged to rvPHEV clades. The effect of these mutations on viral virulence and pathogenesis needs further study. To sum up, the Chinese PHEV strains from other provinces and the 50 PHEV strains obtained in this study are distributed in two subgroups basing on S, M, and N gene sequences, which indicated that the PHEV strains obtained in this study has complex evolutionary trajectories and high genetic diversity.

The Bayesian skyline analysis showed that the effective population size of PHEV has been in a relatively stable state since its discovery, until it expanded sharply around 2015, and then stabilized again, but it is generally higher than the adequate population size before 2015. Unlike PHEV, other porcine coronaviruses, including PEDV and PDCoV, maintained a stable trend in Guangxi province in recent years (Bai et al., 2023; Shi et al., 2024; Li et al., 2024). The accelerated mutation of classical PHEV strains and the emergence of new variants that cause respiratory and diarrhea symptoms are new situations that deserve our high attention (Lorbach et al., 2017; He et al., 2023; Kim et al., 2022; Bahoussi et al., 2022). In this study, the genetic evolution rates of S, M, and N genes were 2.655 × 10^−4^, 5.436 × 10^−4^ and 3.106 × 10^−4^ (substitution/site/year), respectively, indicating M and N genes were more conservative than S gene. In addition, recombination analysis of S gene showed that two PHEV strains existed potential recombination events, indicating genetic diversity of PHEV strains in Guangxi province. Since the relative conservation of M and N gene, and the high diversity of S gene of PHEV (Tables 2, 4), this study focused on the Bayesian time dynamic analysis, amino acid mutation, and recombinant analysis of S gene, but not M and N genes.

Since PHEV was first discovered in Canada in 1957, it has been reported in America, Europe, and Asia (Mora-Díaz et al., 2019; Roe and Alexander, 1958; Quiroga et al., 2008; Rho et al., 2011; Cartwright et al., 1969; Pensaert and Callebaut, 1974; Gao et al., 2011; Dong et al., 2014). PHEV is prevalent all over the world. However, PHEV can only cause encephalitis in piglets, and are subclinical to infected adult pigs. Therefore, PHEV has a relatively small impact on pig herds compared with other pathogenic coronaviruses, so it has not attracted academic attention for a long time. Since the beginning of this century, PHEV has been found and isolated in growing pigs or adult pigs with respiratory and digestive symptoms (Lorbach et al., 2017; He et al., 2023; Kim et al., 2022), so it has been taken more attention recently. The molecular epidemiological study of PHEV in different countries showed that there was great variation in PHEV (Lorbach et al., 2017; He et al., 2023; Kim et al., 2022; Li et al., 2016). Especially, the emergence of rvPHEV is a new phenomenon and might cause serious harm to pig industry, which needs to further study (He et al., 2023). At present, most of the prevalent PHEVs are variants of the original PHEV strain, and there is no specific drug or vaccine for PHEV (Turlewicz-Podbielska and Pomorska-Mól, 2021; Mora-Díaz et al., 2019; Mora-Díaz et al., 2020). The real epidemic situations of PHEV need to be further studied, and the harms of PHEV to pig industry need to be further estimated. Therefore, the surveillance of PHEV, and the genetic and evolutionary analysis were performed in this study, and the results indicated that the prevalent PHEV strains in Guangxi province showed high genetic diversity, which will help to make effective prevention and control measures to reduce losses of this pathogen. To the best of our knowledge, this is the first report on the genetic and evolutionary characteristics of PHEV in southern China.

## 5 Conclusion

Fifty PHEV S, M, and N gene sequences were amplified and sequenced from clinical samples collected from Guangxi province in southern China during 2021–2024. The phylogenetic trees based on PHEV S, M, and N gene sequences revealed that the PHEV strains from different countries could be divided into two groups G1 and G2, and the 50 PHEV strains obtained in this study distributed in subgroup G1c and G2b. The PHEV strains existed variation of mutation, and recombination. The population size of PHEV has been in a relatively stable state since its discovery, until it expanded sharply around 2015, and then stabilized again. The epidemic strains from Guangxi province, southern China showed high genetic diversity.

## Data Availability

The datasets presented in this study can be found in online repositories. The names of the repository/repositories and accession number(s) can be found in the article/Supplementary material.

## Footnotes

^1^ https://www.dnastar.com/software/

^2^ https://blast.ncbi.nlm.nih.gov/Blast.cgi

^3^ https://www.bioedit.com/

^4^ https://www.megasoftware.net/archived_version_active_download

^5^ https://itol.embl.de/

^6^ http://beast.community/

^7^ http://www.bioinf.manchester.ac.uk/recombination/programs

^8^ https://github.com/Stephane-S/Simplot_PlusPlus

## References

[ref1] Alexander T. J. Richards W. P. Roe C. K. (1959). An encephalomyelitis of suckling pigs in Ontario. Can. J. Comp. Med. Vet. Sci. 23, 316–319, PMID: 17649182 PMC1582309

[ref2] Alsop J. E. (2006). A presumptive case of vomiting and wasting disease in a swine nucleus herd. J. Swine Health Product. 14, 97–100. doi: 10.54846/jshap/459

[ref3] Armstrong J. Niemann H. Smeekens S. Rottier P. Warren G. (1984). Sequence and topology of a model intracellular membrane protein, E1 glycoprotein, from a coronavirus. Nature 308, 751–752. doi: 10.1038/308751a0, PMID: 6325918 PMC7095125

[ref4] Arndt A. L. Larson B. J. Hogue B. G. (2010). A conserved domain in the coronavirus membrane protein tail is important for virus assembly. J. Virol. 84, 11418–11428. doi: 10.1128/jvi.01131-10, PMID: 20719948 PMC2953170

[ref5] Bahoussi A. N. Guo Y. Y. Shi R. Z. Wang P. H. Li Y. Q. Wu C. X.. (2022). Genetic characteristics of porcine hemagglutinating encephalomyelitis coronavirus: identification of naturally occurring mutations between 1970 and 2015. Front. Microbiol. 13:860851. doi: 10.3389/fmicb.2022.860851, PMID: 35369458 PMC8971845

[ref6] Bai J. Du C. Lu Y. Wang R. Su X. Yu K.. (2023). Phylogenetic and spatiotemporal analyses of porcine epidemic diarrhea virus in Guangxi, China during 2017-2022. Animals 13:1215. doi: 10.3390/ani13071215, PMID: 37048471 PMC10093014

[ref7] Beniac D. R. Andonov A. Grudeski E. Booth T. F. (2006). Architecture of the SARS coronavirus prefusion spike. Nat. Struct. Mol. Biol. 13, 751–752. doi: 10.1038/nsmb1123, PMID: 16845391 PMC7097490

[ref8] Boniotti M. B. Papetti A. Lavazza A. Alborali G. Sozzi E. Chiapponi C.. (2016). Porcine epidemic diarrhea virus and discovery of a recombinant swine enteric coronavirus, Italy. Emerg. Infect. Dis. 22, 83–87. doi: 10.3201/eid2201.150544 26689738 PMC4696687

[ref9] Bosch B. J. van der Zee R. de Haan C. A. Rottier P. J. (2003). The coronavirus spike protein is a class I virus fusion protein: structural and functional characterization of the fusion core complex. J. Virol. 77, 8801–8811. doi: 10.1128/jvi.77.16.8801-8811.2003, PMID: 12885899 PMC167208

[ref10] Cagliani R. Forni D. Clerici M. Sironi M. (2020). Computational inference of selection underlying the evolution of the novel coronavirus, severe acute respiratory syndrome coronavirus 2. J. Virol. 94, e00411–e00420. doi: 10.1128/jvi.00411-20 32238584 PMC7307108

[ref11] Cartwright S. F. Lucas M. Cavill J. P. Gush A. F. Blandford T. B. (1969). Vomiting and wasting disease of piglets. Vet. Rec. 84, 175–176. doi: 10.1136/vr.84.7.175 5813276

[ref12] Cong Y. Ulasli M. Schepers H. Mauthe M. V'Kovski P. Kriegenburg F.. (2020). Nucleocapsid protein recruitment to replication-transcription complexes plays a crucial role in coronaviral life cycle. J. Virol. 94, e01925–e01919. doi: 10.1128/jvi.01925-19, PMID: 31776274 PMC6997762

[ref13] Cornelissen L. A. Wierda C. M. van der Meer F. J. Herrewegh A. A. Horzinek M. C. Egberink H. F.. (1997). Hemagglutinin-esterase, a novel structural protein of torovirus. J. Virol. 71, 5277–5286. doi: 10.1128/jvi.71.7.5277-5286.1997, PMID: 9188596 PMC191764

[ref14] Cutlip R. C. Mengeling W. L. (1972). Lesions induced by hemagglutinating encephalomyelitis virus strain 67N in pigs. Am. J. Vet. Res. 33, 2003–2009, PMID: 5074702

[ref15] Dong B. Gao W. Lu H. Zhao K. Ding N. Liu W.. (2015). A small region of porcine hemagglutinating encephalomyelitis virus spike protein interacts with the neural cell adhesion molecule. Intervirology 58, 130–137. doi: 10.1159/000381060, PMID: 25925196 PMC7179542

[ref16] Dong B. Lu H. Zhao K. Liu W. Gao W. Lan Y.. (2014). Identification and genetic characterization of porcine hemagglutinating encephalomyelitis virus from domestic piglets in China. Arch. Virol. 159, 2329–2337. doi: 10.1007/s00705-014-2070-y, PMID: 24756345 PMC7087033

[ref17] Emmott E. Munday D. Bickerton E. Britton P. Rodgers M. A. Whitehouse A.. (2013). The cellular interactome of the coronavirus infectious bronchitis virus nucleocapsid protein and functional implications for virus biology. J. Virol. 87, 9486–9500. doi: 10.1128/jvi.00321-13, PMID: 23637410 PMC3754094

[ref18] Fan W. Chen J. Zhang Y. Deng Q. Wei L. Zhao C.. (2022). Phylogenetic and spatiotemporal analyses of the complete genome sequences of avian coronavirus infectious bronchitis virus in China during 1985-2020: revealing coexistence of multiple transmission chains and the origin of LX4-type virus. Front. Microbiol. 13:693196. doi: 10.3389/fmicb.2022.693196, PMID: 35444624 PMC9013971

[ref19] Gao W. He W. Zhao K. Lu H. Ren W. Du C.. (2010). Identification of NCAM that interacts with the PHE-CoV spike protein. Virol. J. 7, 254–264. doi: 10.1186/1743-422x-7-254, PMID: 20863409 PMC2955716

[ref20] Gao W. Zhao K. Zhao C. Du C. Ren W. Song D.. (2011). Vomiting and wasting disease associated with hemagglutinating encephalomyelitis viruses infection in piglets in Jilin. China. Virol. J. 8, 130–138. doi: 10.1186/1743-422x-8-130, PMID: 21418610 PMC3071789

[ref21] Gorkhali R. Koirala P. Rijal S. Mainali A. Baral A. Bhattarai H. K. (2021). Structure and function of major SARS-CoV-2 and SARS-CoV proteins. Bioinform. Biol. Insights 15:11779322211025876. doi: 10.1177/11779322211025876, PMID: 34220199 PMC8221690

[ref22] He W. T. Li D. Baele G. Zhao J. Jiang Z. Ji X.. (2023). Newly identified lineages of porcine hemagglutinating encephalomyelitis virus exhibit respiratory phenotype. Virus Evol. 9, 1–11. doi: 10.1093/ve/vead051, PMID: 37711483 PMC10499004

[ref23] Hirano N. Nomura R. Tawara T. Tohyama K. (2004). Neurotropism of swine haemagglutinating encephalomyelitis virus (coronavirus) in mice depending upon host age and route of infection. J. Comp. Pathol. 130, 58–65. doi: 10.1016/s0021-9975(03)00083-5, PMID: 14693125 PMC7127506

[ref24] Hu X. Feng S. Shi K. Shi Y. Yin Y. Long F.. (2023). Development of a quadruplex real-time quantitative RT-PCR for detection and differentiation of PHEV, PRV, CSFV, and JEV. Front. Vet. Sci. 10:1276505. doi: 10.3389/fvets.2023.1276505, PMID: 38026635 PMC10643766

[ref25] Huang Q. Yu L. Petros A. M. Gunasekera A. Liu Z. Xu N.. (2004). Structure of the N-terminal RNA-binding domain of the SARS CoV nucleocapsid protein. Biochemistry 43, 6059–6063. doi: 10.1021/bi036155b 15147189

[ref26] Kim Y. Lee K. M. Jang G. Lee C. (2022). Complete genome sequence of a novel porcine hemagglutinating encephalomyelitis virus strain identified in South Korea. Arch. Virol. 167, 1381–1385. doi: 10.1007/s00705-022-05414-w, PMID: 35397684 PMC8994818

[ref27] Letunic I. Bork P. (2021). Interactive tree of life (iTOL) v5: an online tool for phylogenetic tree display and annotation. Nucleic Acids Res. 49, W293–W296. doi: 10.1093/nar/gkab301, PMID: 33885785 PMC8265157

[ref28] Li B. Gao Y. Ma Y. Shi K. Shi Y. Feng S.. (2024). Genetic and evolutionary analysis of porcine deltacoronavirus in Guangxi province, southern China, from 2020 to 2023. Microorganisms 12:416. doi: 10.3390/microorganisms12020416, PMID: 38399820 PMC10893222

[ref29] Li Z. He W. Lan Y. Zhao K. Lv X. Lu H.. (2016). The evidence of porcine hemagglutinating encephalomyelitis virus induced nonsuppurative encephalitis as the cause of death in piglets. PeerJ 4, e2443–e2461. doi: 10.7717/peerj.2443, PMID: 27672502 PMC5028786

[ref30] Li S. Lin L. Wang H. Yin J. Ren Y. Zhao Z.. (2003). The epitope study on the SARS-CoV nucleocapsid protein. Genom. Proteom. Bioinform. 1, 198–206. doi: 10.1016/s1672-0229(03)01025-8, PMID: 15629032 PMC5172353

[ref31] Llanes A. Restrepo C. M. Caballero Z. Rajeev S. Kennedy M. A. Lleonart R. (2020). Betacoronavirus genomes: how genomic information has been used to deal with past outbreaks and the COVID-19 pandemic. Int. J. Mol. Sci. 21:4546. doi: 10.3390/ijms21124546, PMID: 32604724 PMC7352669

[ref32] Lorbach J. N. Wang L. Nolting J. M. Benjamin M. G. Killian M. L. Zhang Y.. (2017). Porcine hemagglutinating encephalomyelitis virus and respiratory disease in exhibition swine, Michigan, USA, 2015. Emerg. Infect. Dis. 23, 1168–1171. doi: 10.3201/eid2307.170019, PMID: 28628449 PMC5512476

[ref33] McBride R. van Zyl M. Fielding B. C. (2014). The coronavirus nucleocapsid is a multifunctional protein. Viruses 6, 2991–3018. doi: 10.3390/v6082991, PMID: 25105276 PMC4147684

[ref34] Mengeling W. L. Cutlip R. C. (1972). Experimentally induced infection of newborn pigs with hemagglutinating encephalomyelitis virus strain 67N. Am. J. Vet. Res. 33, 953–956, PMID: 4553997

[ref35] Meyniel-Schicklin L. de Chassey B. André P. Lotteau V. (2012). Viruses and interactomes in translation. Mol. Cell. Proteomics 11, M111.014738-1–M111.014738-12. doi: 10.1074/mcp.M111.014738 PMC3394946 22371486

[ref36] Mitchell D. Corner A. H. Bannister G. L. Greig A. S. (1961). Studies on pathogenic porcine enteroviruses: 1. Preliminary investigations. Can. J. Comp. Med. Vet. Sci. 25, 85–93, PMID: 17649295 PMC1583158

[ref37] Mora-Díaz J. C. Magtoto R. Houston E. Baum D. Carrillo-Ávila J. A. Temeeyasen G.. (2020). Detecting and monitoring porcine hemagglutinating encephalomyelitis virus, an underresearched betacoronavirus. mSphere 5, e00199–e00120. doi: 10.1128/mSphere.00199-20, PMID: 32376700 PMC7203454

[ref38] Mora-Díaz J. C. Piñeyro P. E. Houston E. Zimmerman J. Giménez-Lirola L. G. (2019). Porcine hemagglutinating encephalomyelitis virus: a review. Front. Vet. Sci. 6, 53–64. doi: 10.3389/fvets.2019.00053, PMID: 30873421 PMC6402421

[ref39] Mora-Díaz J. C. Piñeyro P. E. Rauh R. Nelson W. Sankoh Z. Gregg E.. (2021). Porcine hemagglutinating encephalomyelitis virus infection in vivo and ex vivo. J. Virol. 95, e02335–e02320. doi: 10.1128/jvi.02335-20, PMID: 33762411 PMC8316118

[ref40] Neuman B. W. Kiss G. Kunding A. H. Bhella D. Baksh M. F. Connelly S.. (2011). A structural analysis of M protein in coronavirus assembly and morphology. J. Struct. Biol. 174, 11–22. doi: 10.1016/j.jsb.2010.11.021, PMID: 21130884 PMC4486061

[ref41] Pensaert M. B. Callebaut P. E. (1974). Characteristics of a coronavirus causing vomition and wasting in pigs. Arch. Gesamte Virusforsch. 44, 35–50. doi: 10.1007/bf01242179, PMID: 4823866 PMC7087132

[ref42] Quiroga M. A. Cappuccio J. Piñeyro P. Basso W. Moré G. Kienast M.. (2008). Hemagglutinating encephalomyelitis coronavirus infection in pigs Argentina. Emerg. Infect Dis. 14, 484–486. doi: 10.3201/eid1403.070825, PMID: 18325268 PMC2570804

[ref43] Rambaut A. Lam T. T. Max Carvalho L. Pybus O. G. (2016). Exploring the temporal structure of heterochronous sequences using TempEst (formerly path-O-gen). Virus Evol. 2:vew007. doi: 10.1093/ve/vew007, PMID: 27774300 PMC4989882

[ref44] Rho S. Moon H. J. Park S. J. Kim H. K. Keum H. O. Han J. Y.. (2011). Detection and genetic analysis of porcine hemagglutinating encephalomyelitis virus in South Korea. Virus Genes 42, 90–96. doi: 10.1007/s11262-010-0551-y, PMID: 21103919 PMC7089545

[ref45] Roe C. K. Alexander T. J. (1958). A disease of nursing pigs previously unreported in Ontario. Can. J. Comp. Med. Vet. Sci. 22, 305–307. 17649076 PMC1614651

[ref46] Sasseville A. M. Boutin M. Gélinas A. M. Dea S. (2002). Sequence of the 3′-terminal end (8.1 kb) of the genome of porcine haemagglutinating encephalomyelitis virus: comparison with other haemagglutinating coronaviruses. J. Gen. Virol. 83, 2411–2416. doi: 10.1099/0022-1317-83-10-2411, PMID: 12237422

[ref47] Shi K. Li B. Shi Y. Feng S. Yin Y. Long F.. (2024). Phylogenetic and evolutionary analysis of porcine epidemic diarrhea virus in Guangxi province, China, during 2020 and 2024. Viruses 16:1126. doi: 10.3390/v16071126, PMID: 39066288 PMC11281377

[ref48] Shi J. Zhao K. Lu H. Li Z. Lv X. Lan Y.. (2018). Genomic characterization and pathogenicity of a porcine hemagglutinating encephalomyelitis virus strain isolated in China. Virus Genes 54, 672–683. doi: 10.1007/s11262-018-1591-y, PMID: 30078094 PMC7089186

[ref49] Siah A. Breyta R. B. Warheit K. I. Gagne N. Purcell M. K. Morrison D.. (2020). Genomes reveal genetic diversity of piscine orthoreovirus in farmed and free-ranging salmonids from Canada and USA. Virus Evol. 6:veaa054. doi: 10.1093/ve/veaa054, PMID: 33381304 PMC7751156

[ref50] Stohlman S. A. Baric R. S. Nelson G. N. Soe L. H. Welter L. M. Deans R. J. (1988). Specific interaction between coronavirus leader RNA and nucleocapsid protein. J. Virol. 62, 4288–4295. doi: 10.1128/jvi.62.11.4288-4295.1988, PMID: 2845141 PMC253863

[ref51] Sun W. Shi Z. Wang P. Zhao B. Li J. Wei X.. (2023). Metavirome analysis reveals a high prevalence of porcine hemagglutination encephalomyelitis virus in clinically healthy pigs in China. Pathogens 12, 510–526. doi: 10.3390/pathogens12040510, PMID: 37111396 PMC10144687

[ref52] Tang T. K. Wu M. P. Chen S. T. Hou M. H. Hong M. H. Pan F. M.. (2005). Biochemical and immunological studies of nucleocapsid proteins of severe acute respiratory syndrome and 229E human coronaviruses. Proteomics 5, 925–937. doi: 10.1002/pmic.200401204, PMID: 15759315 PMC7167620

[ref53] Tsai C. C. Chou C. H. Wang H. V. Ko Y. Z. Chiang T. Y. Chiang Y. C. (2015). Biogeography of the Phalaenopsis amabilis species complex inferred from nuclear and plastid DNAs. BMC Plant Biol. 15:202. doi: 10.1186/s12870-015-0560-z, PMID: 26276316 PMC4537552

[ref54] Turlewicz-Podbielska H. Pomorska-Mól M. (2021). Porcine coronaviruses: overview of the state of the art. Virol. Sin. 36, 833–851. doi: 10.1007/s12250-021-00364-0, PMID: 33723809 PMC7959302

[ref55] Wong N. A. Saier M. H. Jr. (2021). The SARS-coronavirus infection cycle: a survey of viral membrane proteins, their functional interactions and pathogenesis. Int. J. Mol. Sci. 22, 1308–1373. doi: 10.3390/ijms22031308 33525632 PMC7865831

[ref56] Woo P. C. Y. Lau S. K. P. Choi G. K. Y. Huang Y. Sivakumar S. Tsoi H. W.. (2016). Molecular epidemiology of canine picornavirus in Hong Kong and Dubai and proposal of a novel genus in Picornaviridae. Infect. Genet. Evol. 41, 191–200. doi: 10.1016/j.meegid.2016.03.033 27051044

[ref57] Yagami K. Izumi Y. Kajiwara N. Sugiyama F. Sugiyama Y. (1993). Neurotropism of mouse-adapted haemagglutinating encephalomyelitis virus. J. Comp. Pathol. 109, 21–27. doi: 10.1016/s0021-9975(08)80237-x, PMID: 8408778 PMC7130299

[ref58] Zhang J. Xiao T. Cai Y. Chen B. (2021). Structure of SARS-CoV-2 spike protein. Curr. Opin. Virol. 50, 173–182. doi: 10.1016/j.coviro.2021.08.010, PMID: 34534731 PMC8423807

[ref59] Zhao Y. Zhang H. Zhao J. Zhong Q. Jin J. H. Zhang G. Z. (2016). Evolution of infectious bronchitis virus in China over the past two decades. J. Gen. Virol. 97, 1566–1574. doi: 10.1099/jgv.0.000464, PMID: 27008625 PMC7079583

