# Supplementary material for: Genetic diversity and evolution of porcine hemagglutinating encephalomyelitis virus in Guangxi province of China during 2021–2024
Source: Front Microbiol. 2024 Oct 9;15:1474552. doi: 10.3389/fmicb.2024.1474552 (PMC11496168; doi:10.3389/fmicb.2024.1474552)
Supplement: Supplementary file 1 [file Data_Sheet_1.pdf]

Supplementary Materials

Supplementary Table S1 The information on S gene of PHEV strains used in this study

Accession NO.	Strain	Origin	Date	Accession NO.	Strain	Origin	Date
AY078417	67N	USA	1962	PP646298	PHEV-GXYL2021-01	Yulin, GX, CHN	Dec, 2021
MW165134	PHEV/67N/US/1970	USA	1970	PP646299	PHEV-GXYL2021-02	Yulin, GX, CHN	Dec, 2021
AF481863	IAF-404	CAN	1999	PP646300	PHEV-GXYL2021-03	Yulin, GX, CHN	Dec, 2021
DQ011855	VW572	BEL	/	PP646301	PHEV-GXYL2021-04	Yulin, GX, CHN	Dec, 2021
EU919227	HEV-JT06	CAN	/	PP646302	PHEV-GXNN2021-01	Nanning, GX, CHN	Dec, 2021
KY492680	UU	NLD	Apr, 2015	PP646303	PHEV-GXNN2022-01	Nanning, GX, CHN	Jan, 2022
OL542832	GNU-2113	KOR	Jan, 2021	PP646304	PHEV-GXNN2022-02	Nanning, GX, CHN	Jan, 2022
KY419107	USA/15TOSU1209/2015	USA	Jul, 2015	PP646305	PHEV-GXNN2022-03	Nanning, GX, CHN	Aug, 2022
KY419113	USA-15TOSU1582	USA	Jul, 2015	PP646306	PHEV-GXNN2022-04	Nanning, GX, CHN	Nov, 2022
KY419104	USA/15TOSU0331/2015	USA	Aug, 2015	PP646307	PHEV-GXNN2022-05	Nanning, GX, CHN	Nov, 2022
KY419105	USA/15TOSU0582/2015	USA	Aug, 2015	PP646308	PHEV-GXNN2022-06	Nanning, GX, CHN	Dec, 2022
KY419106	USA/15TOSU1785/2015	USA	Aug, 2015	PP646309	PHEV-GXNN2022-07	Nanning, GX, CHN	Dec, 2022
KY419109	USA/15TOSU1655/2015	USA	Aug, 2015	PP646310	PHEV-GXNN2022-08	Nanning, GX, CHN	Dec, 2022
KY419110	USA/15TOSU1582/2015	USA	Aug, 2015	PP646311	PHEV-GXNN2022-09	Nanning, GX, CHN	Dec, 2022
KY419111	USA/15TOSU1727/2015	USA	Aug, 2015	PP646312	PHEV-GXNN2022-10	Nanning, GX, CHN	Dec, 2022
KY419112	USA/15TOSU1765/2015	USA	Aug, 2015	PP646313	PHEV-GXNN2022-11	Nanning, GX, CHN	Dec, 2022
KY419103	USA/15TOSU25049/2015	USA	Sep, 2015	PP646314	PHEV-GXCZ2022-01	Chongzuo, GX, CHN	Apr, 2022
KY994645	JL/2008	CHN	Aug, 2008	PP646315	PHEV-GXCZ2022-02	Chongzuo, GX, CHN	Dec, 2022

KU127229	HEV-CC14	CHN	Feb, 2014	PP646316	PHEV-GXCZ2022-03	Chongzuo, GX, CHN	Dec, 2022
MF083115	CC14	CHN	May, 2014	PP646317	PHEV-GXNN2023-01	Nanning, GX, CHN	Jan, 2023
OQ305205	PHEV/GD/2017	CHN	Jun, 2017	PP646318	PHEV-GXNN2023-02	Nanning, GX, CHN	Jan, 2023
OQ305206	PHEV/HLJ/2017	CHN	Jun, 2017	PP646319	PHEV-GXNN2023-03	Nanning, GX, CHN	Mar, 2023
OQ305207	PHEV/SC/2017	CHN	Jun, 2017	PP646320	PHEV-GXNN2023-04	Nanning, GX, CHN	Jul, 2023
OQ305208	PHEV/ZJ/2017	CHN	Jun, 2017	PP646321	PHEV-GXNN2023-05	Nanning, GX, CHN	Jul, 2023
OP959790	LJ/2021	CHN	Dec, 2021	PP646322	PHEV-GXNN2023-06	Nanning, GX, CHN	Jul, 2023
OQ798806	rvPHEV17	CHN	Dec, 2018	PP646323	PHEV-GXNN2023-07	Nanning, GX, CHN	Jul, 2023
OQ798807	rvPHEV1	CHN	Apr, 2021	PP646324	PHEV-GXNN2023-08	Nanning, GX, CHN	Aug, 2023
OQ798808	rvPHEV10	CHN	May, 2021	PP646325	PHEV-GXNN2023-09	Nanning, GX, CHN	Sep, 2023
OQ798809	rvPHEV13	CHN	May, 2021	PP646326	PHEV-GXNN2023-10	Nanning, GX, CHN	Sep, 2023
OQ798810	rvPHEV14	CHN	Nov, 2019	PP646327	PHEV-GXNN2023-11	Nanning, GX, CHN	Dec, 2023
OQ798811	rvPHEV15	CHN	Dec, 2019	PP646328	PHEV-GXHZ2023-01	Hezhou, GX, CHN	Feb, 2023
OQ798812	rvPHEV16	CHN	Jan, 2020	PP646329	PHEV-GXHZ2023-02	Hezhou, GX, CHN	Feb, 2023
OQ798813	rvPHEV18	CHN	May, 2021	PP646330	PHEV-GXHZ2023-03	Hezhou, GX, CHN	Feb, 2023
OQ798814	rvPHEV19	CHN	May, 2021	PP646331	PHEV-GXHZ2023-04	Hezhou, GX, CHN	Dec, 2023
OQ798815	rvPHEV2	CHN	Apr, 2021	PP646332	PHEV-GXHZ2023-05	Hezhou, GX, CHN	Dec, 2023
OQ798816	rvPHEV20	CHN	Apr, 2021	PP646333	PHEV-GXYL2023-01	Yulin, GX, CHN	Apr, 2023
OQ798817	rvPHEV21	CHN	Mar, 2020	PP646334	PHEV-GXYL2023-02	Yulin, GX, CHN	Jul, 2023
OQ798818	rvPHEV22	CHN	Nov, 2021	PP646335	PHEV-GXYL2023-03	Yulin, GX, CHN	Aug, 2023
OQ798819	rvPHEV23	CHN	Nov, 2021	PP646336	PHEV-GXYL2023-04	Yulin, GX, CHN	Aug, 2023
OQ798820	rvPHEV24	CHN	Nov, 2021	PP646337	PHEV-GXYL2023-05	Yulin, GX, CHN	Aug, 2023
OQ798821	rvPHEV25	CHN	Nov, 2021	PP646338	PHEV-GXLB2023-01	Laibin, GX, CHN	May, 2023

OQ798822	rvPHEV26	CHN	Nov, 2021	PP646339	PHEV-GXLB2023-02	Laibin, GX, CHN	May, 2023
OQ798823	rvPHEV3	CHN	Apr, 2021	PP646340	PHEV-GXLB2023-03	Laibin, GX, CHN	May, 2023
OQ798824	rvPHEV4	CHN	Feb, 2019	PP646341	PHEV-GXLB2023-04	Laibin, GX, CHN	Jul, 2023
OQ798825	rvPHEV5	CHN	Mar, 2016	PP646342	PHEV-GXCZ2023-01	Chongzuo, GX, CHN	Jul, 2023
OQ798826	rvPHEV6	CHN	May, 2020	PP646343	PHEV-GXBS2023-01	Baise, GX, CHN	Oct, 2023
OQ798827	rvPHEV7	CHN	Apr, 2019	PP646344	PHEV-GXBS2023-02	Baise, GX, CHN	Oct, 2023
OQ798828	rvPHEV8	CHN	Mar, 2020	PP646345	PHEV-GXBS2023-03	Baise, GX, CHN	Oct, 2023
OQ798829	rvPHEV9	CHN	Nov, 2019	PP646346	PHEV-GXNN2024-01	Nanning, GX, CHN	Jan, 2024
OQ798832	rvPHEV11	CHN	Mar, 2020	PP646347	PHEV-GXNN2024-02	Nanning, GX, CHN	Jan, 2024
OQ798833	rvPHEV12	CHN	Jan, 2020				

Note: CHN: China; USA: the United States of America; CAN: Canada; CZE: Czech Republic; KOR: Korea; NLD: Netherlands; BEL: Belgium; GX: Guangxi province of China. The sequences obtained in this study are marked with red. The same as follows.

Supplementary Table S2 The information on M gene of PHEV strains used in this study

Accession NO.	Strain	Origin	Date	Accession NO.	Strain	Origin	Date
AY078417	67N	USA	1962	PP646348	PHEV-GXYL2021-01	Yulin, GX, CHN	Dec, 2021
MW165134	PHEV/67N/US/1970	USA	1970	PP646349	PHEV-GXYL2021-02	Yulin, GX, CHN	Dec, 2021
AF481863	IAF-404	CAN	1999	PP646350	PHEV-GXYL2021-03	Yulin, GX, CHN	Dec, 2021
DQ011855	VW572	BEL	/	PP646351	PHEV-GXYL2021-04	Yulin, GX, CHN	Dec, 2021
OL542832	GNU-2113	KOR	Jan, 2021	PP646352	PHEV-GXNN2021-01	Nanning, GX, CHN	Dec, 2021
KY419107	USA/15TOSU1209/2015	USA	Jul, 2015	PP646353	PHEV-GXNN2022-01	Nanning, GX, CHN	Jan, 2022
KY419113	USA-15TOSU1582	USA	Jul, 2015	PP646354	PHEV-GXNN2022-02	Nanning, GX, CHN	Jan, 2022
KY419104	USA/15TOSU0331/2015	USA	Aug, 2015	PP646355	PHEV-GXNN2022-03	Nanning, GX, CHN	Aug, 2022
KY419105	USA/15TOSU0582/2015	USA	Aug, 2015	PP646356	PHEV-GXNN2022-04	Nanning, GX, CHN	Nov, 2022
KY419106	USA/15TOSU1785/2015	USA	Aug, 2015	PP646357	PHEV-GXNN2022-05	Nanning, GX, CHN	Nov, 2022
KY419109	USA/15TOSU1655/2015	USA	Aug, 2015	PP646358	PHEV-GXNN2022-06	Nanning, GX, CHN	Dec, 2022
KY419110	USA/15TOSU1582/2015	USA	Aug, 2015	PP646359	PHEV-GXNN2022-07	Nanning, GX, CHN	Dec, 2022
KY419111	USA/15TOSU1727/2015	USA	Aug, 2015	PP646360	PHEV-GXNN2022-08	Nanning, GX, CHN	Dec, 2022
KY419112	USA/15TOSU1765/2015	USA	Aug, 2015	PP646361	PHEV-GXNN2022-09	Nanning, GX, CHN	Dec, 2022
KY419103	USA/15TOSU25049/2015	USA	Aug, 2015	PP646362	PHEV-GXNN2022-10	Nanning, GX, CHN	Dec, 2022
KY994645	JL/2008	CHN	Aug, 2008	PP646363	PHEV-GXNN2022-11	Nanning, GX, CHN	Dec, 2022
KU127229	HEV-CC14	CHN	Feb, 2014	PP646364	PHEV-GXCZ2022-01	Chongzuo, GX, CHN	Apr, 2022
MF083115	CC14	CHN	May, 2014	PP646365	PHEV-GXCZ2022-02	Chongzuo, GX, CHN	Dec, 2022
FJ009233	HEV-JT06	CHN	/	PP646366	PHEV-GXCZ2022-03	Chongzuo, GX, CHN	Dec, 2022

KY348417	HEV-JL15	CHN	Aug, 2015	PP646367	PHEV-GXNN2023-01	Nanning, GX, CHN	Nov, 2023
OQ305205	PHEV/GD/2017	CHN	Jun, 2017	PP646368	PHEV-GXNN2023-02	Nanning, GX, CHN	Jan, 2023
OQ305206	PHEV/HLJ/2017	CHN	Jun, 2017	PP646369	PHEV-GXNN2023-03	Nanning, GX, CHN	Mar, 2023
OQ305207	PHEV/SC/2017	CHN	Jun, 2017	PP646370	PHEV-GXNN2023-04	Nanning, GX, CHN	Jul, 2023
OQ305208	PHEV/ZJ/2017	CHN	Jun, 2017	PP646371	PHEV-GXNN2023-05	Nanning, GX, CHN	Jul, 2023
OP959790	LJ/2021	CHN	Dec, 2021	PP646372	PHEV-GXNN2023-06	Nanning, GX, CHN	Jul, 2023
OQ798806	rvPHEV17	CHN	Dec, 2018	PP646373	PHEV-GXNN2023-07	Nanning, GX, CHN	Jul, 2023
OQ798807	rvPHEV1	CHN	Apr, 2021	PP646374	PHEV-GXNN2023-08	Nanning, GX, CHN	Aug, 2023
OQ798808	rvPHEV10	CHN	May, 2021	PP646375	PHEV-GXNN2023-09	Nanning, GX, CHN	Sep, 2023
OQ798809	rvPHEV13	CHN	May, 2021	PP646376	PHEV-GXNN2023-10	Nanning, GX, CHN	Sep, 2023
OQ798810	rvPHEV14	CHN	Nov,2019	PP646377	PHEV-GXNN2023-11	Nanning, GX, CHN	Dec, 2023
OQ798811	rvPHEV15	CHN	Dec, 2019	PP646378	PHEV-GXHZ2023-01	Hezhou, GX, CHN	Feb, 2023
OQ798812	rvPHEV16	CHN	Jan, 2020	PP646379	PHEV-GXHZ2023-02	Hezhou, GX, CHN	Feb, 2023
OQ798813	rvPHEV18	CHN	May, 2021	PP646380	PHEV-GXHZ2023-03	Hezhou, GX, CHN	Feb, 2023
OQ798814	rvPHEV19	CHN	May, 2021	PP646381	PHEV-GXHZ2023-04	Hezhou, GX, CHN	Dec, 2023
OQ798815	rvPHEV2	CHN	Apr, 2021	PP646382	PHEV-GXHZ2023-05	Hezhou, GX, CHN	Dec, 2023
OQ798816	rvPHEV20	CHN	Apr, 2021	PP646383	PHEV-GXYL2023-01	Yulin, GX, CHN	Apr, 2023
OQ798817	rvPHEV21	CHN	Mar, 2020	PP646384	PHEV-GXYL2023-02	Yulin, GX, CHN	Jul, 2023
OQ798818	rvPHEV22	CHN	Nov, 2021	PP646385	PHEV-GXYL2023-03	Yulin, GX, CHN	Aug, 2023
OQ798819	rvPHEV23	CHN	Nov, 2021	PP646386	PHEV-GXYL2023-04	Yulin, GX, CHN	Aug, 2023
OQ798821	rvPHEV25	CHN	Nov, 2021	PP646387	PHEV-GXYL2023-05	Yulin, GX, CHN	Aug, 2023
OQ798822	rvPHEV26	CHN	Nov, 2021	PP646388	PHEV-GXLB2023-01	Laibin, GX, CHN	May, 2023
OQ798823	rvPHEV3	CHN	Apr, 2021	PP646389	PHEV-GXLB2023-02	Laibin, GX, CHN	May, 2023

OQ798824	rvPHEV4	CHN	Feb, 2019	PP646390	PHEV-GXLB2023-03	Laibin, GX, CHN	May, 2023
OQ798825	rvPHEV5	CHN	Mar, 2016	PP646391	PHEV-GXLB2023-04	Laibin, GX, CHN	Jul, 2023
OQ798826	rvPHEV6	CHN	May, 2020	PP646392	PHEV-GXCZ2023-01	Chongzuo, GX, CHN	Jul, 2023
OQ798827	rvPHEV7	CHN	Apr, 2019	PP646393	PHEV-GXBS2023-01	Baise, GX, CHN	Oct, 2023
OQ798828	rvPHEV8	CHN	Mar, 2020	PP646394	PHEV-GXBS2023-02	Baise, GX, CHN	Oct, 2023
OQ798829	rvPHEV9	CHN	Nov, 2019	PP646395	PHEV-GXBS2023-03	Baise, GX, CHN	Oct, 2023
				PP646396	PHEV-GXNN2024-01	Nanning, GX, CHN	Jan, 2024
				PP646397	PHEV-GXNN2024-02	Nanning, GX, CHN	Jan, 2024

Supplementary Table S3 The information on N gene of PHEV strains used in this study

Accession NO.	Strain	Origin	Date	Accession NO.	Strain	Origin	Date
AY078417	67N	USA	1962	PP646398	PHEV-GXYL2021-01	Yulin, GX, CHN	Dec, 2021
MW165134	PHEV/67N/US/1970	USA	1970	PP646399	PHEV-GXYL2021-02	Yulin, GX, CHN	Dec, 2021
AF481863	IAF-404	CAN	1999	PP646400	PHEV-GXYL2021-03	Yulin, GX, CHN	Dec, 2021
DQ011855	VW572	BEL	/	PP646401	PHEV-GXYL2021-04	Yulin, GX, CHN	Dec, 2021
OL542832	GNU-2113	KOR	Jan, 2021	PP646402	PHEV-GXNN2021-01	Nanning, GX, CHN	Dec, 2021
KY419107	USA/15TOSU1209/2015	USA	Jul, 2015	PP646403	PHEV-GXNN2022-01	Nanning, GX, CHN	Jan, 2022
KY419113	USA-15TOSU1582	USA	Jul, 2015	PP646404	PHEV-GXNN2022-02	Nanning, GX, CHN	Jan, 2022
KY419104	USA/15TOSU0331/2015	USA	Aug, 2015	PP646405	PHEV-GXNN2022-03	Nanning, GX, CHN	Aug, 2022
KY419105	USA/15TOSU0582/2015	USA	Aug, 2015	PP646406	PHEV-GXNN2022-04	Nanning, GX, CHN	Nov, 2022
KY419106	USA/15TOSU1785/2015	USA	Aug, 2015	PP646407	PHEV-GXNN2022-05	Nanning, GX, CHN	Nov, 2022
KY419109	USA/15TOSU1655/2015	USA	Aug, 2015	PP646408	PHEV-GXNN2022-06	Nanning, GX, CHN	Dec, 2022
KY419110	USA/15TOSU1582/2015	USA	Aug, 2015	PP646409	PHEV-GXNN2022-07	Nanning, GX, CHN	Dec, 2022
KY419111	USA/15TOSU1727/2015	USA	Aug, 2015	PP646410	PHEV-GXNN2022-08	Nanning, GX, CHN	Dec, 2022
KY419112	USA/15TOSU1765/2015	USA	Aug, 2015	PP646411	PHEV-GXNN2022-09	Nanning, GX, CHN	Dec, 2022
KY419103	USA/15TOSU25049/2015	USA	Sep, 2015	PP646412	PHEV-GXNN2022-10	Nanning, GX, CHN	Dec, 2022
MH475347	CZE/P234/2016	CZE	Apr, 2016	PP646413	PHEV-GXNN2022-11	Nanning, GX, CHN	Dec, 2022
MH475349	CZE/P236/2016	CZE	Apr, 2016	PP646414	PHEV-GXCZ2022-01	Chongzuo, GX, CHN	Apr, 2022
MH475350	CZE/P237/2016	CZE	Apr, 2016	PP646415	PHEV-GXCZ2022-02	Chongzuo, GX, CHN	Dec, 2022
MH475351	CZE/P238/2016	CZE	Apr, 2016	PP646416	PHEV-GXCZ2022-03	Chongzuo, GX, CHN	Dec, 2022

MH475352	CZE/P579/2016	CZE	Nov, 2016	PP646417	PHEV-GXNN2023-01	Nanning, GX, CHN	Jan, 2023
MH475353	CZE/P581/2016	CZE	Nov, 2016	PP646418	PHEV-GXNN2023-02	Nanning, GX, CHN	Jan, 2023
MH475354	CZE/P681/2016	CZE	Dec, 2016	PP646419	PHEV-GXNN2023-03	Nanning, GX, CHN	Mar, 2023
MH475355	CZE/P692/2016	CZE	Dec, 2016	PP646420	PHEV-GXNN2023-04	Nanning, GX, CHN	Jul, 2023
MH475356	CZE/P694/2016	CZE	Dec, 2016	PP646421	PHEV-GXNN2023-05	Nanning, GX, CHN	Jul, 2023
KY994645	JL/2008	CHN	Aug, 2008	PP646422	PHEV-GXNN2023-06	Nanning, GX, CHN	Jul, 2023
KU127229	HEV-CC14	CHN	Feb, 2014	PP646423	PHEV-GXNN2023-07	Nanning, GX, CHN	Jul, 2023
MF083115	CC14	CHN	May, 2014	PP646424	PHEV-GXNN2023-08	Nanning, GX, CHN	Aug, 2023
OQ305205	PHEV/GD/2017	CHN	Jun, 2017	PP646425	PHEV-GXNN2023-09	Nanning, GX, CHN	Sep, 2023
OQ305206	PHEV/HLJ/2017	CHN	Jun, 2017	PP646426	PHEV-GXNN2023-10	Nanning, GX, CHN	Sep, 2023
OQ305207	PHEV/SC/2017	CHN	Jun, 2017	PP646427	PHEV-GXNN2023-11	Nanning, GX, CHN	Dec, 2023
OQ305208	PHEV/ZJ/2017	CHN	Jun, 2017	PP646428	PHEV-GXHZ2023-01	Hezhou, GX, CHN	Feb, 2023
OP959790	LJ/2021	CHN	Dec, 2021	PP646429	PHEV-GXHZ2023-02	Hezhou, GX, CHN	Feb, 2023
FJ009234	HEV-JT06	CHN	/	PP646430	PHEV-GXHZ2023-03	Hezhou, GX, CHN	Feb, 2023
OQ798806	rvPHEV17	CHN	Dec, 2018	PP646431	PHEV-GXHZ2023-04	Hezhou, GX, CHN	Dec, 2023
OQ798807	rvPHEV1	CHN	Apr, 2021	PP646432	PHEV-GXHZ2023-05	Hezhou, GX, CHN	Dec, 2023
OQ798808	rvPHEV10	CHN	May, 2021	PP646433	PHEV-GXYL2023-01	Yulin, GX, CHN	Apr, 2023
OQ798809	rvPHEV13	CHN	May, 2021	PP646434	PHEV-GXYL2023-02	Yulin, GX, CHN	Jul, 2023
OQ798811	rvPHEV15	CHN	Dec, 2019	PP646435	PHEV-GXYL2023-03	Yulin, GX, CHN	Aug, 2023
OQ798812	rvPHEV16	CHN	Jan, 2020	PP646436	PHEV-GXYL2023-04	Yulin, GX, CHN	Aug, 2023
OQ798813	rvPHEV18	CHN	May, 2021	PP646437	PHEV-GXYL2023-05	Yulin, GX, CHN	Aug, 2023
OQ798814	rvPHEV19	CHN	May, 2021	PP646438	PHEV-GXLB2023-01	Laibin, GX, CHN	May, 2023
OQ798815	rvPHEV2	CHN	Apr, 2021	PP646439	PHEV-GXLB2023-02	Laibin, GX, CHN	May, 2023

OQ798816	rvPHEV20	CHN	Apr, 2021	PP646440	PHEV-GXLB2023-03	Laibin, GX, CHN	May, 2023
OQ798817	rvPHEV21	CHN	Mar, 2020	PP646441	PHEV-GXLB2023-04	Laibin, GX, CHN	Jul, 2023
OQ798819	rvPHEV23	CHN	Nov, 2021	PP646442	PHEV-GXCZ2023-01	Chongzuo, GX, CHN	Jul, 2023
OQ798820	rvPHEV24	CHN	Nov, 2021	PP646443	PHEV-GXBS2023-01	Baise, GX, CHN	Oct, 2023
OQ798821	rvPHEV25	CHN	Nov, 2021	PP646444	PHEV-GXBS2023-02	Baise, GX, CHN	Oct, 2023
OQ798822	rvPHEV26	CHN	Nov, 2021	PP646445	PHEV-GXBS2023-03	Baise, GX, CHN	Oct, 2023
OQ798823	rvPHEV3	CHN	Apr, 2021	PP646446	PHEV-GXNN2024-01	Nanning, GX, CHN	Jan, 2024
OQ798824	rvPHEV4	CHN	Feb, 2019	PP646447	PHEV-GXNN2024-02	Nanning, GX, CHN	Jan, 2024
OQ798826	rvPHEV6	CHN	May, 2020				
OQ798827	rvPHEV7	CHN	Apr, 2019				
OQ798828	rvPHEV8	CHN	Mar, 2020				
OQ798829	rvPHEV9	CHN	Nov, 2019				

Supplementary Table S4 Detection results of clinical samples

Date	Area	Tissue sample	Positive sample (%)	Nasopharyngeal swab	Positive sample (%)	Total	
						Sample	Positive (%)
2021	Yulin	195	7 (3.59%)	0	0	195	7 (3.59%)
	Nanning	71	1 (1.41%)	0	0	71	1 (1.41%)
2022	Yulin	109	0	0	0	109	0
	Nanning	980	7 (0.71%)	1914	44 (2.30%)	2894	51 (1.76%)
	Chongzuo	127	6 (4.72%)	0	0	127	6 (4.72%)
	Liuzhou	4	0	0	0	4	0
	Guigang	2	0	0	0	2	0
	Beihai	1	0	0	0	1	0
	Fangchenggang	0	0	32	4 (12.50%)	32	4 (12.50%)
	Hezhou	90	1 (1.11%)	32	12 (37.50%)	122	13 (10.66%)
	Qinzhou	8	0	0	0	8	0
	Baise	12	0	0	0	12	0
2023	Yulin	398	19 (4.77%)	0	0	398	19 (4.77%)
	Nanning	249	26 (10.44%)	48	3 (6.25%)	297	29 (9.76%)
	Chongzuo	546	10 (1.83%)	0	0	546	10 (1.83%)
	Liuzhou	31	0	0	0	31	0
	Guigang	198	1 (0.51%)	0	0	198	1 (0.51%)
	Beihai	20	0	0	0	20	0
	Fangchenggang	386	0	0	0	386	0
	Hezhou	24	2 (8.33%)	126	4 (3.17%)	150	6 (4.00%)
	Qinzhou	20	0	0	0	20	0

	Baise	421	6 (1.43%)	19	0	440	6 (1.36%)
	Laibin	80	1 (1.25%)	140	28 (20.00%)	220	29 (13.18%)
	Guilin	56	0	0	0	56	0
	Hechi	0	0	89	4 (4.49%)	89	4 (4.49%)
	Wuzhou	16	0	0	0	16	0
2024	Yulin	16	0	0	0	16	0
	Nanning	176	0	340	10 (2.94%)	516	10 (1.94%)
	Liuzhou	10	0	0	0	10	0
Total	Nanning	1476	34 (2.30%)	2302	57 (2.48%)	3778	91 (2.41%)
	Liuzhou	45	0	0	0	45	0
	Guilin	56	0	0	0	56	0
	Wuzhou	16	0	0	0	16	0
	Beihai	21	0	0	0	21	0
	Yulin	718	26 (3.62%)	0	0	718	26 (3.62%)
	Guigang	200	1 (0.50%)	0	0	200	1 (0.50%)
	Hezhou	114	3 (2.63%)	158	16 (10.13%)	272	19 (6.99%)
	Hechi	0	0	89	4 (4.49%)	89	4 (4.49%)
	Baise	433	6 (1.39%)	19	0	452	6 (1.33%)
	Qinzhou	28	0	0	0	28	0
	Fangchenggang	386	0	32	4 (12.50%)	418	4 (0.96%)
	Chongzuo	673	16 (2.38%)	0	0	673	16 (2.38%)
	Laibin	80	1 (1.25%)	140	28 (20.00%)	220	29 (13.18%)
**Total**		**4246**	**87 (2.05%)**	**2740**	**109 (3.98%)**	**6986**	**196 (2.81%)**

Supplementary figure

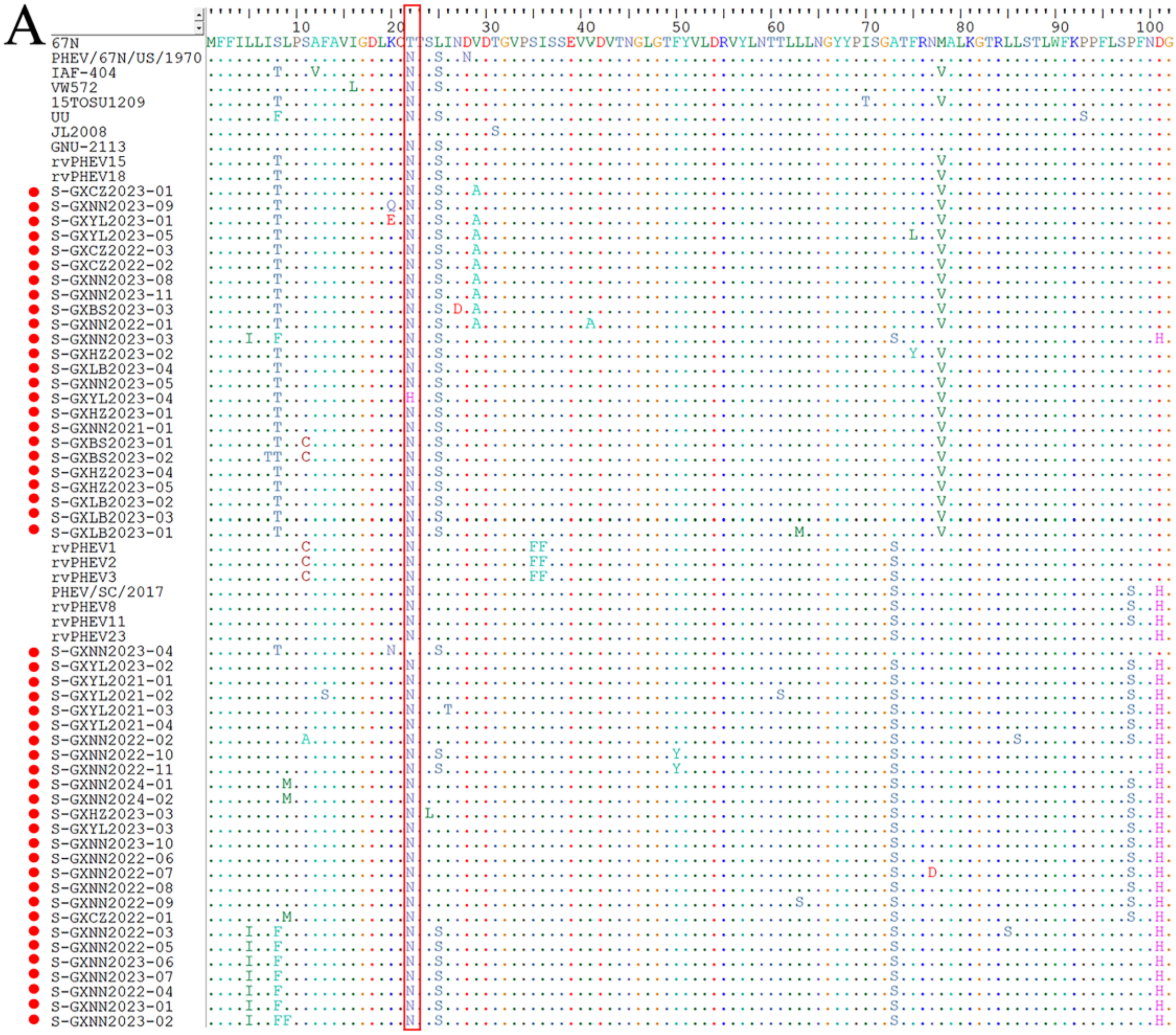

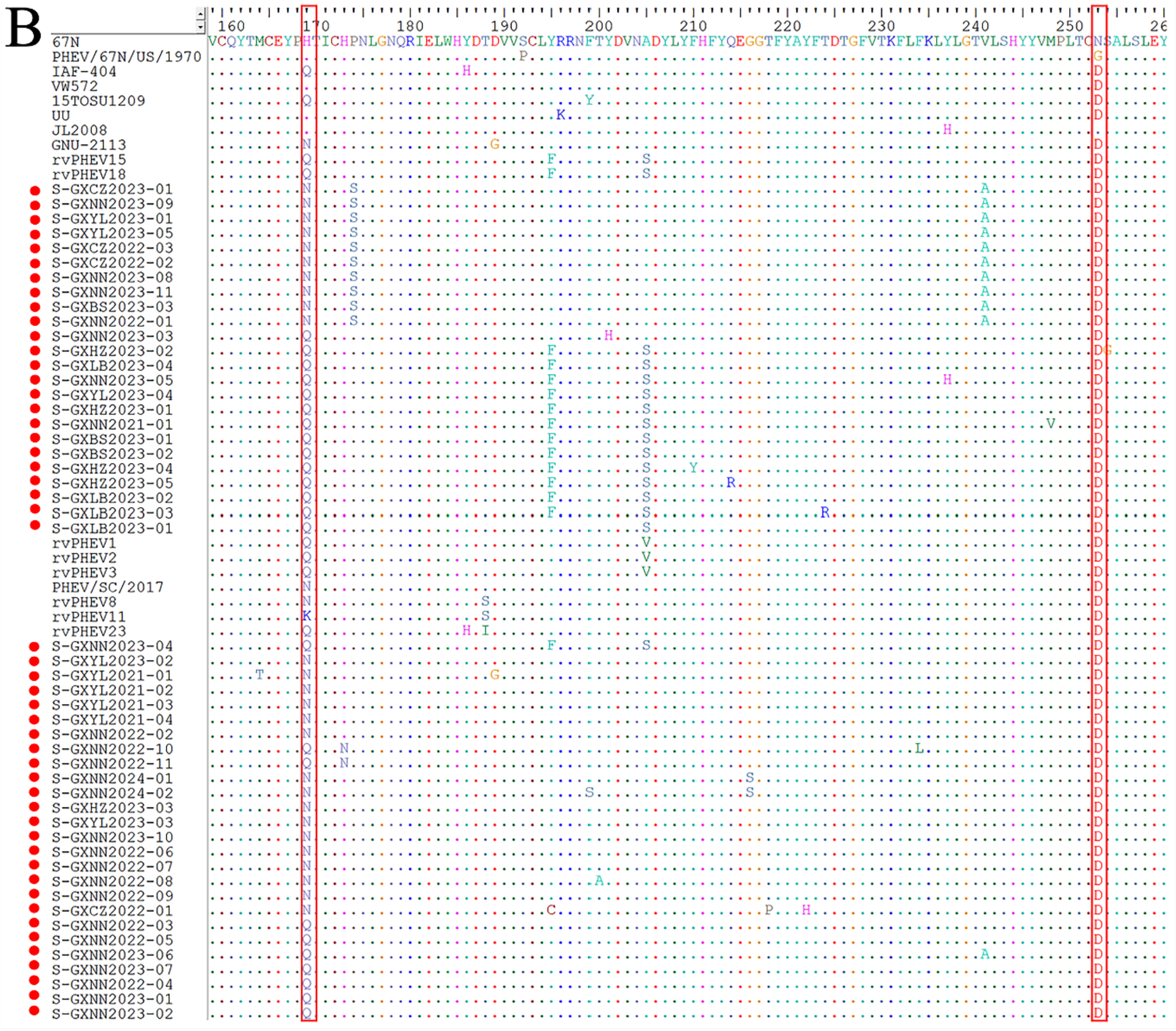

C

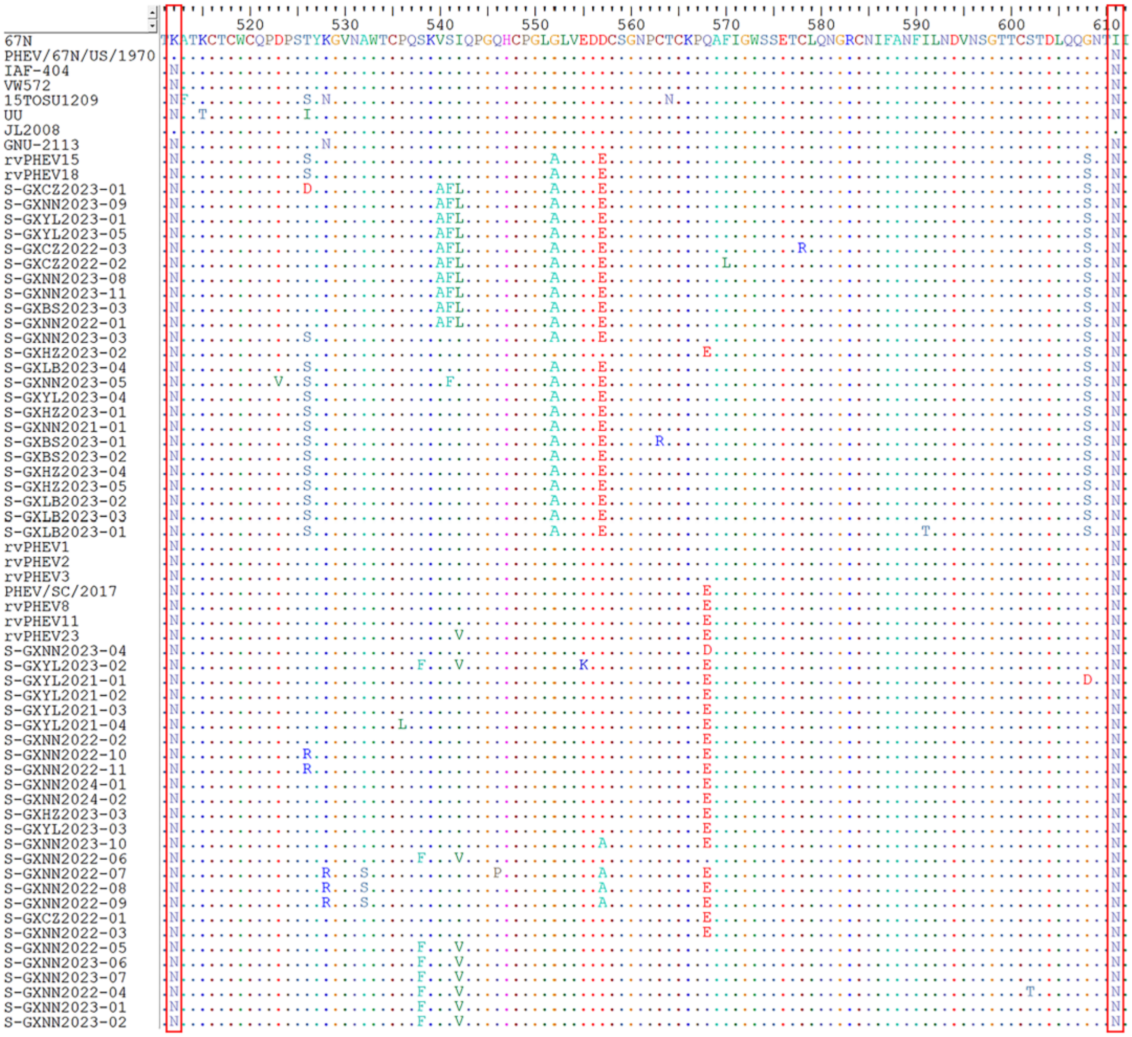

D

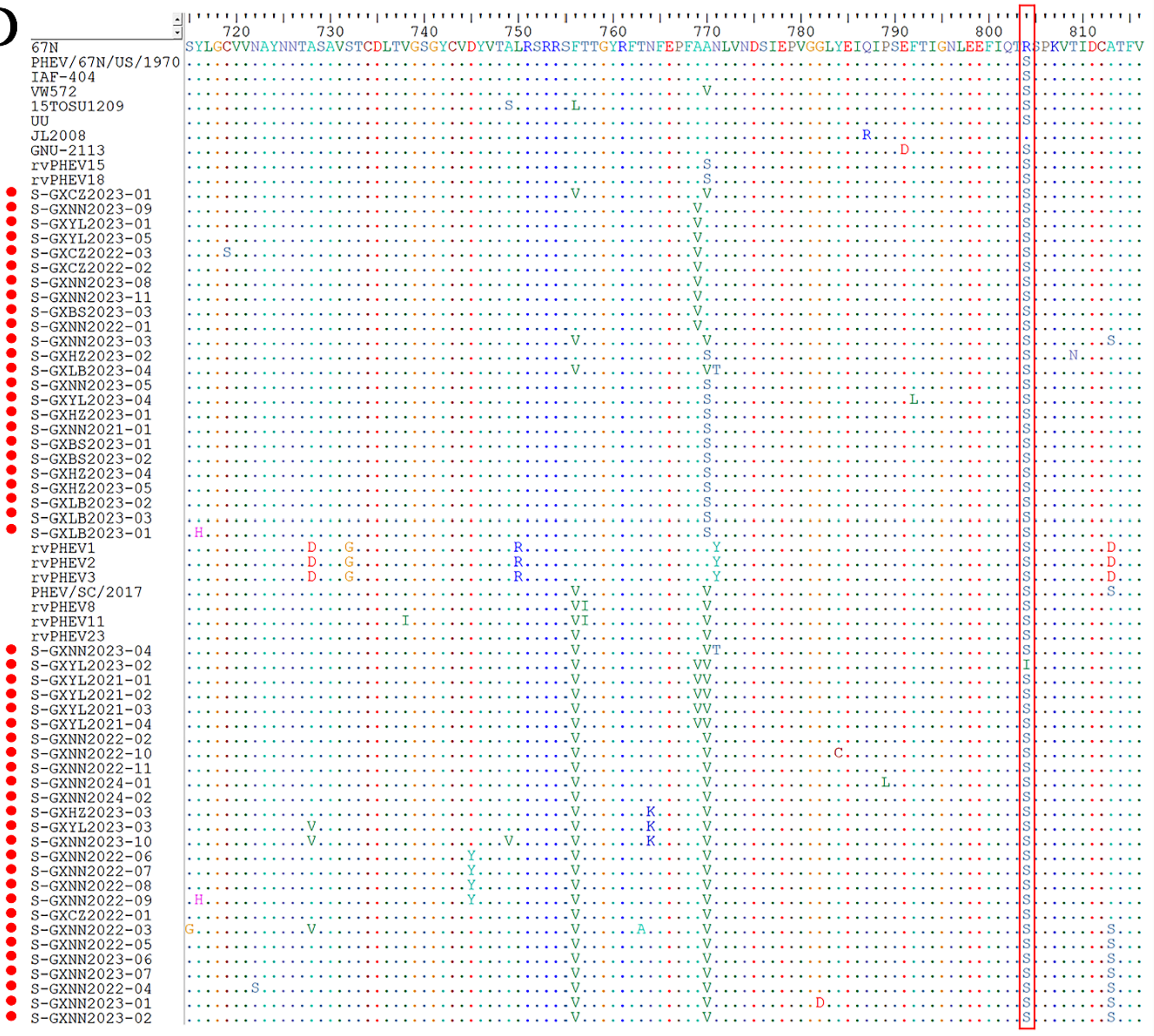
720 730 740 750 760 770 780 790 800 810
67N
SYLGCVVNAYNNTASAVSTCDLTVGSGYCVDYVTALRSRRSFTTGYRFTNFEPFAANLVNDSIEPVGGLYEIQIPSEFTIGNLEEFIQTRSPKVTIDCATFV
PHEV/67N/US/1970
IAF-404
VW572
15TOSU1209
UU
JL2008
GNU-2113
rvPHEV15
rvPHEV18
S-GXCZ2023-01
S-GXNN2023-09
S-GXYL2023-01
S-GXYL2023-05
S-GXCZ2022-03
S-GXCZ2022-02
S-GXNN2023-08
S-GXNN2023-11
S-GXBS2023-03
S-GXNN2022-01
S-GXNN2023-03
S-GXHZ2023-02
S-GXLB2023-04
S-GXNN2023-05
S-GXYL2023-04
S-GXHZ2023-01
S-GXNN2021-01
S-GXBS2023-01
S-GXBS2023-02
S-GXHZ2023-04
S-GXHZ2023-05
S-GXLB2023-02
S-GXLB2023-03
S-GXLB2023-01
rvPHEV1
rvPHEV2
rvPHEV3
PHEV/SC/2017
rvPHEV8
rvPHEV11
rvPHEV23
S-GXNN2023-04
S-GXYL2023-02
S-GXYL2021-01
S-GXYL2021-02
S-GXYL2021-03
S-GXYL2021-04
S-GXNN2022-02
S-GXNN2022-10
S-GXNN2022-11
S-GXNN2024-01
S-GXNN2024-02
S-GXHZ2023-03
S-GXYL2023-03
S-GXNN2023-10
S-GXNN2022-06
S-GXNN2022-07
S-GXNN2022-08
S-GXNN2022-09
S-GXCZ2022-01
S-GXNN2022-03
S-GXNN2022-05
S-GXNN2023-06
S-GXNN2023-07
S-GXNN2022-04
S-GXNN2023-01
S-GXNN2023-02

E

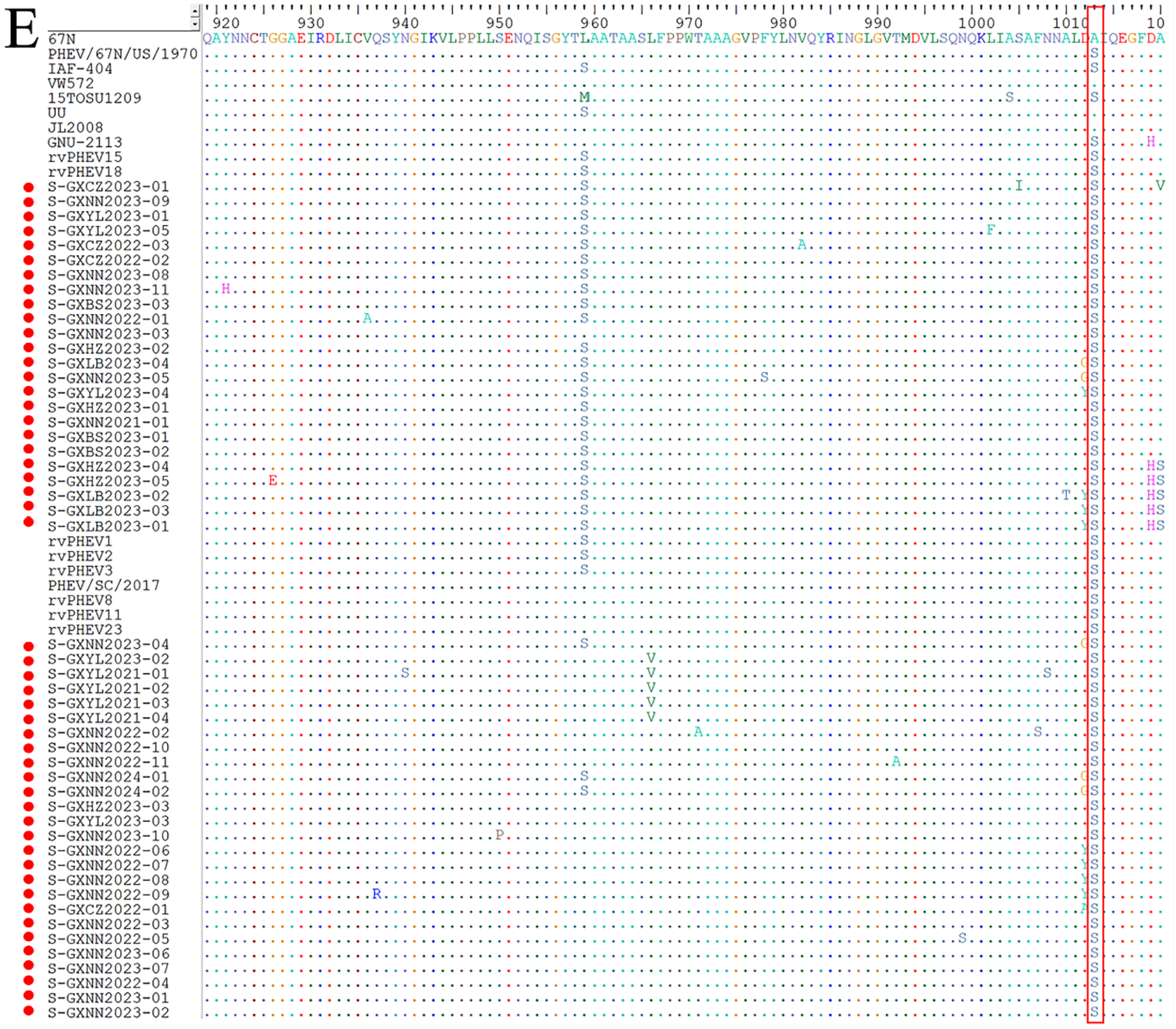

F

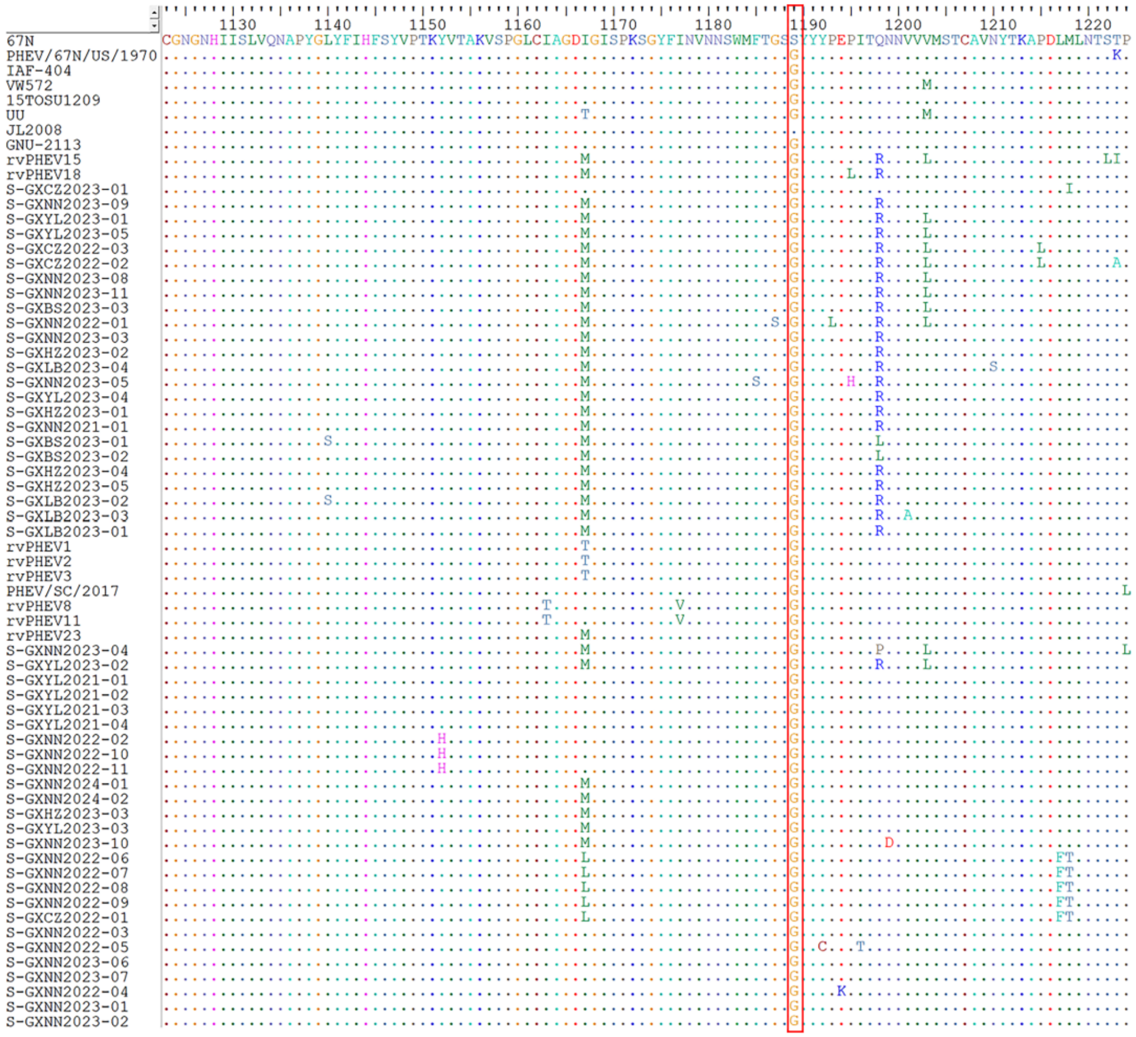

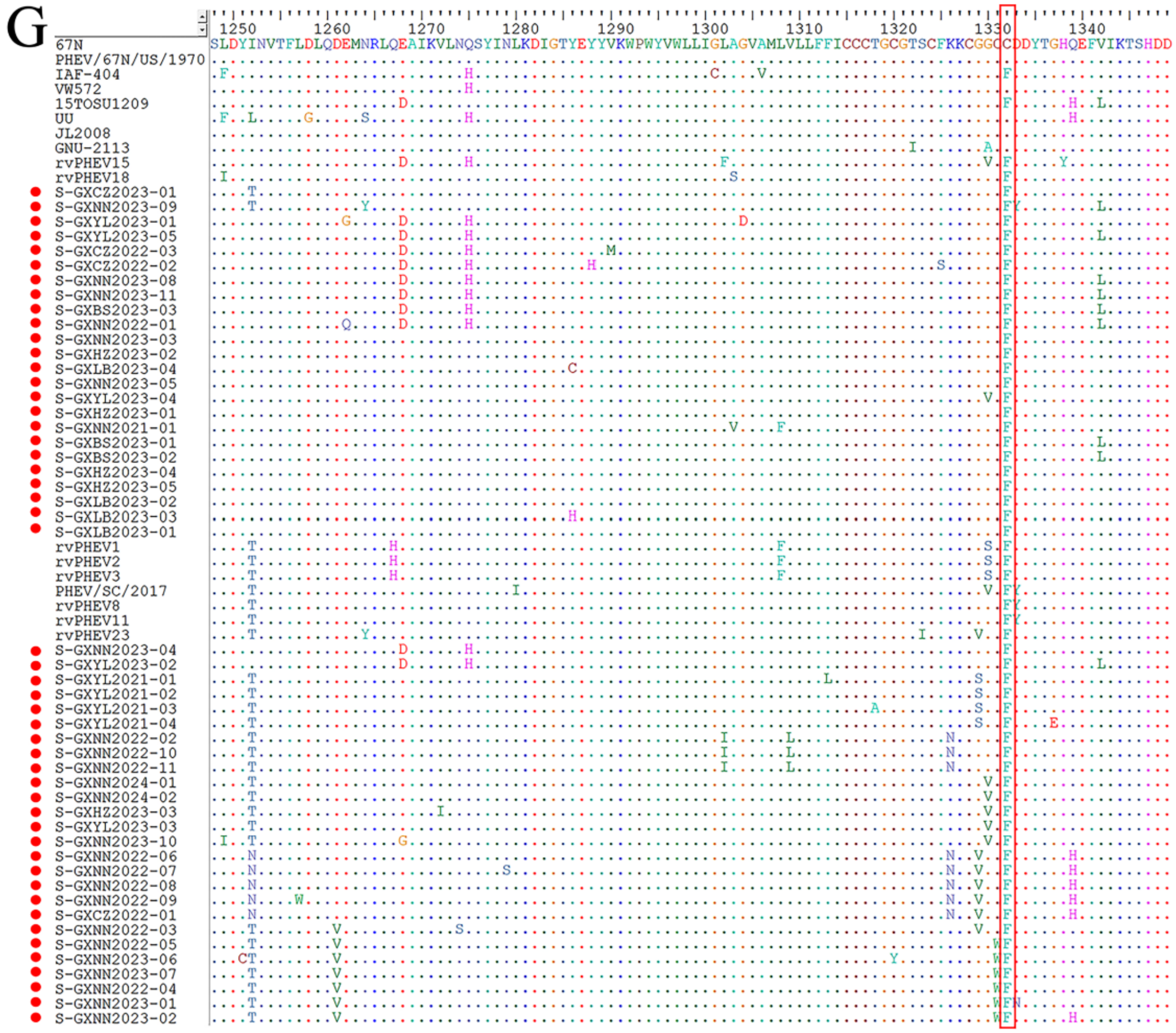

Supplementary Figure S1

Multiple amino acid comparison of PHEV S gene (A-G). The sequences obtained in this study are marked with red spots.
